# Supplementary material for: A systematic comparison of optogenetic approaches to visual restoration
Source: Mol Ther Methods Clin Dev. 2022 Mar 7;25:111–23. doi: 10.1016/j.omtm.2022.03.003 (PMC8956963; doi:10.1016/j.omtm.2022.03.003)
Supplement: Document S1. Tables S1–S5, Figures S1, and Supplemental methods [file mmc1.pdf]

OMTM, Volume *25*

Supplemental information

A systematic comparison of optogenetic approaches to visual restoration

Michael J. Gilhooley, Moritz Lindner, Teele Palumaa, Steven Hughes, Stuart N. Peirson, and Mark W. Hankins

Supplemental Materials

Methods

Mice

The following origin lines were bred together to reach the desired genotypes.

Table S1 - Genetic background of the various origin lines contributing to the two multi-transgenic lines described. The righthand column refers to genes in which mutations are commonly found in the background strain – not necessarily the particular line used

Origin Line	Genetic Background Strain	Common visually significant mutations[1]
$Pde6b^{rd1/rd12}$[4]	C3H/HeN	$Gpr179^{nob/nob}$, $Pde6b^{rd1/rd1}$
$Opn4^{tm1Yau}$[5]	C57BL/6J	Normal vision
Tg(L7-Cre)[6]	FVB	$Pde6b^{rd1/rd1}$
Tg(grk4-Cre)[7]	C57BL/6J	Normal vision

Experimental mice were of the genotype:

1. Tg(*L7.Cre*)$^{+/-}$; $Pde6b^{rd1/rd1}$; $Opn4^{tm1yau/tm1yau}$
2. Tg(*Grk4.Cre*)$^{+/-}$; $Pde6b^{rd1/rd1}$; $Opn4^{tm1yau/tm1yau}$

As the insertion locus of the Cre transgene in both lines is not known, breeding pairs were maintained as hemizygous x wild type pairs to ensure hemizygous offspring. All mice were genotyped to ensure they were wild type for the endemic $Gpr179^{nob/nob}$ mutation.

Genotyping

Genotypes of individual mice were determined using polymerase chain reaction (PCR) protocols. DNA was extracted from mouse ear clips using sodium hydroxide solution, neutralised with Trizma - HCl. Samples were then incubated with a PCR master mix and primers as described in Table S2 according to manufacturer's instructions. Protocols using Immored® (Bioline, London, UK) ($Pde6b^{rd1}$, *Tg(Cre),* $Gpr179^{nob1}$) were then run on a 2% agarose gel incorporating 4 μmol/l ethidium bromide (Sigma Aldrich, Gillingham, UK) in Tris-acetate-EDTA (TEA) buffer (Severn Biotech, Kidderminster, UK) at 125volts for 25 minutes. SYBR Green® protocols ($Opn4^{+}$ & $Opn4^{tm1Yau}$ (LacZ)) underwent a melt curve protocol with the characteristic product melt temperature used to confirm the amplification of the specific product.

Confirmation of the retinal phenotype (IHC)

The expected retinal degenerate phenotype was confirmed on microscopy of retinal sections, demonstrating an absence of photoreceptor layer in both lines. The expected $Opn4^{-/-}$ phenotype was confirmed by replacement of mouse OPN4 immunoreactivity in the ganglion cell layer with immunoreactivity for β-galactosidase (encoded by *LacZ*) in the same distribution. Cre recombinase

activity allowed appropriate expression of floxed viral protein in both the L7.Cre and Grk4.Cre model (see Figure 1); confirming the expected Cre phenotype.

Table S2 Primers and reaction parameters for polymerase chain reaction based genotyping of mice.

Allele	Primer	Sequence	Product size or product melt tempterature Tm (bp or ^{o}C)	
Pde6b ^{rd1}	1	TGACAATTACTCCTTTTCCCTCAGTCT		
	2	GTAAACAGCAAGAGGCTTTATTGGGAA	*Pde6b* ^{rd1}	550
	3	GCATTAATTCTGGGGCGCATG	*Pde6b* $^{+}$ (wildtype)	400
Tg(Cre)	F	GCGGTCTGGCAGTAAAAACTATC		
	R	GTGAAACAGCATTGCTGTCACTT		
	F (control)	CGTAGGCCACAGAATTGAAAGATCT	Tg(Cre)	101
	R (control)	GTAGGTGGAAATTCTAGCATCATCC	Control	324
Gpr179 ^{nob}	1	TGTGCCTGGGTATCTGTTGA		
	2	GCATGTGCCAAGGGTATCTT	*Gpr179* ^{nob}	400
	3	GCTTACACACTTACACACAGATAGATG	*Gpr179* $^{+}$ (wildtype)	113
Opn4 ^{tm1Yau} (LacZ)	F	AAGCAGTCAGCAGCCCAAAG		
	R	TGTCACCCTCCTGGTCTTGC	*Opn4* ^{tm1Yau} (LacZ)	86°C
Opn4 $^{+}$	F	AGAAGTGGCTTTGGGGA		
	R	GCAGAAGGCATAGAACTCG	*Opn4* $^{+}$	80.2^{o}C

AAV production

The CBA.hOPN4 and (floxed) EF1α-hOPN4 construct plasmids were produced in our laboratory as has been described previously[8, 9]. The (floxed) hSyn.DIO.ReaChR construct was a kind gift from Roger Tsein (R.I.P.) (Addgene plasmid # 50954; http://n2t.net/addgene:50954 ; RRID:Addgene_50954) [10]. These plasmids were used, along with a quad mutant capsid (Y-F)[11] and helper (pAdDF6) plasmids to produce adenoassociated virus according to previously published methods[12].

The titre of DNase1-treated virus was determined by real-time quantitative PCR using the standard curve method designed to amplify a portion of the inverted terminal repeat sequence (ITR) as previously described (Primers: forward: 5'-GGA ACC CCT AGT GAT GGA GTT; reverse: 5'-CGG CCT CAG TGA GCG A)[13].

Intravitreal injections

Anaesthesia was induced and maintained in mice via inhalation of isoflurane (Zoetis, Walton Oaks, UK), Guttatae (gt.) 0.5% proxymethocaine & gt. 1% tropicamide (Minnims brand, Bausch & Lomb U.K Limited, Kingston-Upon-Thames) were applied for topical anaesthesia and to dilate the pupil. Gt. Povidone Iodine 5% (Minims brand, Bausch & Lomb U.K Limited, Kingston-Upon-Thames) was added, before Viscotears (Bausch & Lomb U.K Limited, Kingston-Upon-Thames) and a 2mm round cover slip were applied to the cornea view the posterior segment. A 35-gauge, bevelled needle (NF35BV), on a 10μl NanoFil syringe (both World Precision Instruments, Hitchin, UK) was introduced anterior to the gripping forceps. The needle was advanced into the vitreous, under direct visualisation and 1.5μl of undiluted virus (CBA.hOPN4 4.43x10^{12} viral genomes ml^{-1}, floxed.hOPN4 1.72x10^{12} $vgml^{-1}$, floxed.ReaChR 1.5x10^{13} $vgml^{-1}$, L7-6.hOPN4 7.33x$10^{12}$$vgml^{-1}$) or 0.9% saline was then injected. The needle was held in place for 5 to 10 seconds before a slow withdrawal in order to minimise reflux.

Tissue collection & Immunohistochemistry of retinal flat mounts

Animals were culled by cervical dislocation, eyes immediately enucleated and transferred to 4% PFA for twenty-four hours before dissection under a microscope (EZ4D, Leica, Wetzlar, Germany. Permeabilisation was with Triton X-100 1% in PBS, followed by three ten-minute washes in PBS. A blocking step followed with 10% serum from the animal in which the secondary antibody was raised (Table S4) diluted in 1% Triton X-100 for sixty minutes. Slides were then incubated for three days with a primary antibody solution at the stated concentration (Table S3) diluted in 1% Triton X-100 with

2.5% serum from the animal in which the secondary antibody was raised (Table S4). Three washes of thirty minutes in 0.1% Tween-20 were undertaken before incubation overnight with a secondary antibody solution at a concentration of 1:250 in 1% Triton X-100 - PBS (Table S4). Three washes of thirty minutes in 0.1% Tween-20 were undertaken, followed by a 30-minute incubation with 1:30,000 (circa. 300nmol/l) 4',6-Diamidino-2-Phenylindole, Dihydrochloride (DAPI) (ThermoFisher, Loughborough,UK) and a final wash for 30 minutes in ddH_2O. Slides were then dried and mounted with ProLong® Diamond mounting media (ThermoFisher, Loughborough, UK).
Flat mounts were visualised using a confocal microscope (LSM710; Zeiss, Oberkochen, Germany). Whole retina views of flat mounts were achieved by stitching smaller, overlapping images. Images were then processed to using Fiji-ImageJ®[14].

Table S3 - Primary antibodies used

Target	Target species	Manufacturer	Product Code	Host Animal	Dilution used
Melanopsin	Mouse	Advanced Targeting Systems	uf006 - UF006	Rabbit	1:1,000
Melanopsin	Human	Santa Cruz	sc32870	Rabbit	1:1,000
Green Fluorescent Protein	n/a	Aves	gfp1020	Chicken	1:1000
β-galactasdase	n/a	Abcam	ab9361	Chicken	1:500
L7	Mouse	Santa Cruz	sc137064	Mouse	1:1,000

Table S4 - Secondary antibodies used

Name	Target species	Host Animal	Excitation λ (nm)	Emission λ (nm)	Manufacturer	Product Code	Dilution used
Alexa®-568	Rabbit	Donkey	578	603	invitrogen	a10042	1:250
Alexa®-568	Chicken	Goat	578	603	invitrogen	ag175472	1:250
Alexa®-568	Mouse	Donkey	578	603	invitrogen	a10037	1:250
Alexa®-488	Rabbit	Donkey	495	519	invitrogen	a21206	1:250
Alexa®-488	Mouse	Donkey	495	519	invitrogen	a2102	1:250
Alexa®-488	Chicken	Donkey	495	519	Jackson Immunoresearch	T03-545-155	1:250
DAPI	n/a	n/a	358	461	Thermofisher Scientific	D1306	$300nmoll^{-1}$

Multiple Electrode Array Analysis

Retinal ganglion cell action potential firing rate was extracted in 1 second bins using the MC Rack software from filtered data, with an action potential spike defined as a deviation greater than three standard deviations from the mean baseline noise. This data was then transferred to an Excel spreadsheet template (Modified from the work of S Hughes[15]) for further processing.

Proportion of electrodes showing light responses

Table S5 - Proportion of responsive electrodes; Supplemental to figure 2c

Group	CBA.hOPN4	L7.hOPN4	Grik4.hOPN4	L7.ReaChR	Grik4.hOPN4	L7 Saline	Grik4 Saline
Retinae (N=)	5	6	6	11	5	8	8
Responsive (n=)	55	84	87	92	75	7*	6*
Total	300	360	360	660	300	480	480
Proportion	18%	23%	24%	14%	25%	1%	1%

A light responsive electrode was defined as one with a mean rise of 10Hz in spike firing rate, maintained for 10 seconds (rolling 10 second bins) when compared to mean baseline firing rate in the 10 seconds preceding the stimulus (Table S5). Unresponsive electrodes were excluded from further analysis. In the saline sham injection control group, 1% of electrodes were scored as responsive by this metric: all seven of these individual records were manually reviewed (5 of them being adjacent electrodes on the same retina) and did not represent light responses, but spurious readings (noise), that were not present of repeat trials and were so excluded from further analysis. Similarly, six electrodes (five adjacent on the same retina) were scored responsive in the saline treated Grk4.Cre group but seen on manual review to similarly represent noise and were excluded from analysis.

Irradiance Response Curve Fitting

Responses (maximum firing rate post stimulus) at each of 7 intensities (circa 10^{10} to 10^{16} photons cm^{-2} s^{-1}) were normalised to the response at the intensity with the largest response. A macro, written in visual basic and using MS® Excel's solver module (modified from the work of S Hughes & J Rodgers[9, 15, 16]) using an iterative non-linear regression process, was used to fit a sigmoidal curve to this normalised data by minimising the sum of squares of the regression[17]. The Hill Slope & EC_{50} value was allowed to vary in order to achieve the best fit [18]. Starting values in both cases were defined as an EC_{50} = 13 and Hill slope = 1, as plausible values for photopigments[18]. In both cases, only electrodes where a fit of r^2>0.8 were included in reported mean EC_{50} or used in comparison of curves between groups in the main manuscript. IRC plots of Individual EC50 and Hill slope values for each electrode are shown in figure S1.

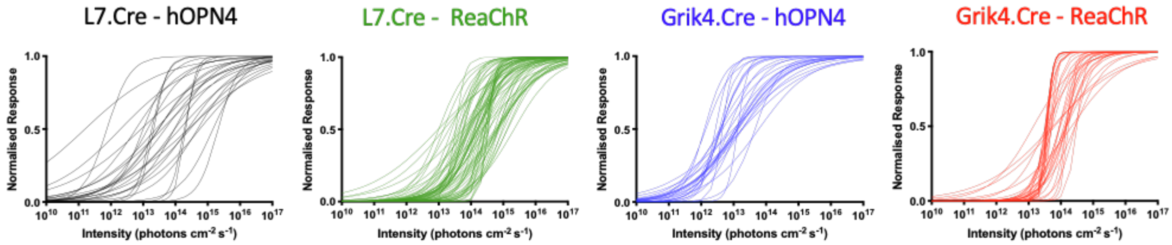

Figure 1 (supplementary) - Plots of IRCs fitted to individual electrodes plotted per treatment group.

Response Decay kinetics

Response decay kinetics, recorded as the total response half life, $t_{1/2}$, was taken as the time interval between the maximum spike firing rate above baseline recorded within 10 seconds of stimulus onset and the point at which the spike firing rate was half this value. Sub-indices were also analysed: spike adaptation half-life was taken as the time interval between the maximum spike firing rate recorded while the stimulus was present and the point at which the firing rate was halfway between this value and that at stimulus offset. True offset half-life was calculated as the time interval between the firing rate at stimulus offset and the time at which the firing rate decayed to half this level. Only values from responsive electrodes with IRC fits r^2>0.8 were included in the mean values that were compared between groups.

Supplemental References

1. Peirson, SN, Brown, LA, Pothecary, CA, Benson, LA, and Fisk, AS (2017). Light and the laboratory mouse. *J Neurosci Methods*.
2. KEELER, C (1966). Retinal degeneration in the mouse is rodless retina. *Journal of Heredity* **57**: 47-50.
3. Keeler, CE (1924). The inheritance of a retinal abnormality in white mice. *Proceedings of the National Academy of Sciences of the United States of America* **10**: 329.
4. Pittler, SJ, and Baehr, W (1991). Identification of a nonsense mutation in the rod photoreceptor cGMP phosphodiesterase beta-subunit gene of the rd mouse. *Proc Natl Acad Sci U S A* **88**: 8322-8326.
5. Hattar, S, Liao, HW, Takao, M, Berson, DM, and Yau, KW (2002). Melanopsin-containing retinal ganglion cells: architecture, projections, and intrinsic photosensitivity. *Science* **295**: 1065-1070.
6. Silvia Marino, PK, Carly Leung, Hetty A. G. M. van der Korput, Jan Trapman, Isabelle Camenisch, Anton Berns and Sebastian Brandner (2002). PTEN is essential for cell migration but not for fate determination and tumourigenesis in the cerebellum. *Development* **129**: 3513-3522.
7. Nakazawa, K, Quirk, MC, Chitwood, RA, Watanabe, M, Yeckel, MF, Sun, LD*, et al.* (2002). Requirement for hippocampal CA3 NMDA receptors in associative memory recall. *Science* **297**: 211-218.
8. De Silva, SR, Barnard, AR, Hughes, S, Tam, SKE, Martin, C, Singh, MS*, et al.* (2017). Long-term restoration of visual function in end-stage retinal degeneration using subretinal human melanopsin gene therapy. *Proc Natl Acad Sci U S A* **114**: 11211-11216.
9. Rodgers, J, Hughes, S, Pothecary, CA, Brown, LA, Hickey, DG, Peirson, SN*, et al.* (2018). Defining the impact of melanopsin missense polymorphisms using in vivo functional rescue. *Hum Mol Genet*.
10. Lin, JY, Knutsen, PM, Muller, A, Kleinfeld, D, and Tsien, RY (2013). ReaChR: a red-shifted variant of channelrhodopsin enables deep transcranial optogenetic excitation. *Nature neuroscience* **16**: 1499-1508.
11. Petrs-Silva, H, Dinculescu, A, Li, Q, Min, SH, Chiodo, V, Pang, JJ*, et al.* (2009). High-efficiency transduction of the mouse retina by tyrosine-mutant AAV serotype vectors. *Mol Ther* **17**: 463-471.
12. de Silva, SR, McClements, ME, Hankins, MW, and MacLaren, RE (2015). Adeno-Associated Viral Gene Therapy for Retinal Disorders. *Gene Delivery and Therapy for Neurological Disorders*: 203-228.
13. Aurnhammer, C, Haase, M, Muether, N, Hausl, M, Rauschhuber, C, Huber, I*, et al.* (2012). Universal real-time PCR for the detection and quantification of adeno-associated virus serotype 2-derived inverted terminal repeat sequences. *Hum Gene Ther Methods* **23**: 18-28.
14. Schindelin, J, Arganda-Carreras, I, Frise, E, Kaynig, V, Longair, M, Pietzsch, T*, et al.* (2012). Fiji: an open-source platform for biological-image analysis. *Nature methods* **9**: 676.

15. Hughes, S (2017). An automaded program for MEA analysis written in MS Visual Basic & MS Excel.
16. Kemmer, G, and Keller, S (2010). Nonlinear least-squares data fitting in Excel spreadsheets. *Nat Protoc* **5**: 267-281.
17. Peirson, SN, Thompson, S, Hankins, MW, and Foster, RG (2005). Mammalian photoentrainment: results, methods, and approaches. *Methods in enzymology*, vol. 393. Elsevier. pp 697-726.
18. Govardovskii, VI, Fyhrquist, N, Reuter, TOM, Kuzmin, DG, and Donner, K (2000). In search of the visual pigment template. *Visual Neuroscience* **17**: 509-528.
