# Supplementary material for: A systematic comparison of optogenetic approaches to visual restoration
Source: Mol Ther Methods Clin Dev. 2022 Mar 7;25:111–23. doi: 10.1016/j.omtm.2022.03.003 (PMC8956963; doi:10.1016/j.omtm.2022.03.003)
Supplement: Document S2. Article plus supplemental information [file mmc2.pdf]

Molecular Therapy
Methods & Clinical Development
Original Article

A systematic comparison of optogenetic approaches to visual restoration

Michael J. Gilhooley,[1,2,3,4] Moritz Lindner,[1,2,5] Teele Palumaa,[2,6] Steven Hughes,[1,2] Stuart N. Peirson,[1,2] and Mark W. Hankins[1,2]

[1]Nuffield Laboratory of Ophthalmology, Nuffield Department of Clinical Neuroscience, University of Oxford, Oxford OX1 3QU, UK; [2]Jules Thorne SCNi, Nuffield Department of Clinical Neuroscience, University of Oxford, Oxford OX1 3QU, UK; [3]The Institute of Ophthalmology, University College London, 11-43 Bath Street, London EC1V 9EL, UK; [4]Moorfields Eye Hospital, 162, City Road, London EC1V 2PD, UK; [5]Institute of Physiology and Pathophysiology, Department of Neurophysiology, Philipps University, Deutschhausstrasse 1-2, Marburg 35037, Germany; [6]East Tallinn Central Hospital Eye Clinic, Ravi 18, 10138 Tallinn, Estonia

During inherited retinal degenerations (IRDs), vision is lost due to photoreceptor cell death; however, a range of optogenetic tools have been shown to restore light responses in animal models. Restored response characteristics vary between tools and the neuronal cell population to which they are delivered: the interplay between these is complex, but targeting upstream neurons (such as retinal bipolar cells) may provide functional benefit by retaining intraretinal signal processing. In this study, our aim was to compare two optogenetic tools: mammalian melanopsin (hOPN4) and microbial red-shifted channelrhodopsin (ReaChR) expressed within two subpopulations of surviving cells in a degenerate retina. Intravitreal adeno-associated viral vectors and mouse models utilising the Cre/lox system restricted expression to populations dominated by bipolar cells or retinal ganglion cells and was compared with non-targeted delivery using the chicken beta actin (CBA) promoter. In summary, we found bipolar-targeted optogenetic tools produced faster kinetics and flatter intensity-response relationships compared with non-targeted or retinal-ganglion-cell-targeted hOPN4. Hence, optogenetic tools of both mammalian and microbial origins show advantages when targeted to bipolar cells. This demonstrates the advantage of bipolar-cell-targeted optogenetics for vision restoration in IRDs. We therefore developed a bipolar-cell-specific gene delivery system employing a compressed promoter with the potential for clinical translation.

INTRODUCTION

Inherited retinal degenerations (IRDs) represent one of the largest causes of visual morbidity in the working age population, affecting around in 1 in 4,000 people.[1] Vision is ultimately lost in these conditions through a final common pathway of photoreceptor loss, driven by a mutation in one of hundreds of different genes known to cause IRDs.[1] The development of a clinical retinal gene replacement therapy based on an adeno-associated viral (AAV) delivery system[2] has heightened the drive to develop mutation-independent approaches to restoring vision, which may be applicable even after photoreceptors are lost. Optogenetics, the introduction of transgenic protein tools rendering targeted cells photosensitive, has been demonstrated in preclinical models to restore light responses using a variety of optogenetic tools[3–15] and has entered early-phase clinical trials (ClinicalTrials.gov: NCT02556736 and NCT03326336; clinicaltrials.gov). Indeed, Sahel et al.[16] recently provided the first description of human visual responses being restored using an optogenetic approach with the case report[16] of a participant in the PIONEER study. In this phase I/II study, an optogenetic tool (Chrimson R) is delivered using intravitreal injection of AAV into patients with end-stage IRD. Following treatment, the patient was able to perform visually guided tasks with occipital electroencephalograms (EEGs) recorded in response to visual stimuli. The full results of this clinical trial are still to be published at the time of writing.

This trial illustrates two important choices that must be made in designing an optogenetic therapeutic: the first is what tool to use? In PIONEER, Chrimson R was employed due to its advantageous spectral sensitivity and the fact it is only active above ambient-light intensities (a useful safety feature for early clinical trials as it is not constitutively active). This, however, requires cumbersome amplifying googles, so it may not be foremost for therapeutic translation in the longer term.

The second choice is which cell types should the tool be targeted to? In PIONEER, a high efficiency, non-specific (CAG) promoter is used with the aim of expressing good quantities of optogenetic tool in as many cell types as possible (ensuring as high a magnitude light response as possible). However, reports in animal models have emerged demonstrating that useful intraretinal signaling processing can be retained when upstream retinal neurons are targeted specifically: in particular, that ON- and OFF-type responses can be restored by targeting populations including retinal bipolar cells.[8,12,13,17–19] Beyond intraretinal signal processing, the cellular environment of the transduced cell type will impact how a particular optogenetic

Received 16 December 2021; accepted 4 March 2022;
https://doi.org/10.1016/j.omtm.2022.03.003.

Correspondence: Mark W. Hankins, Nuffield Laboratory of Ophthalmology, Nuffield Department of Clinical Neuroscience, University of Oxford, Oxford OX1 3QU, UK.
E-mail: mark.hankins@eye.ox.ac.uk

This is an open access article under the CC BY license (http://creativecommons.org/licenses/by/4.0/).

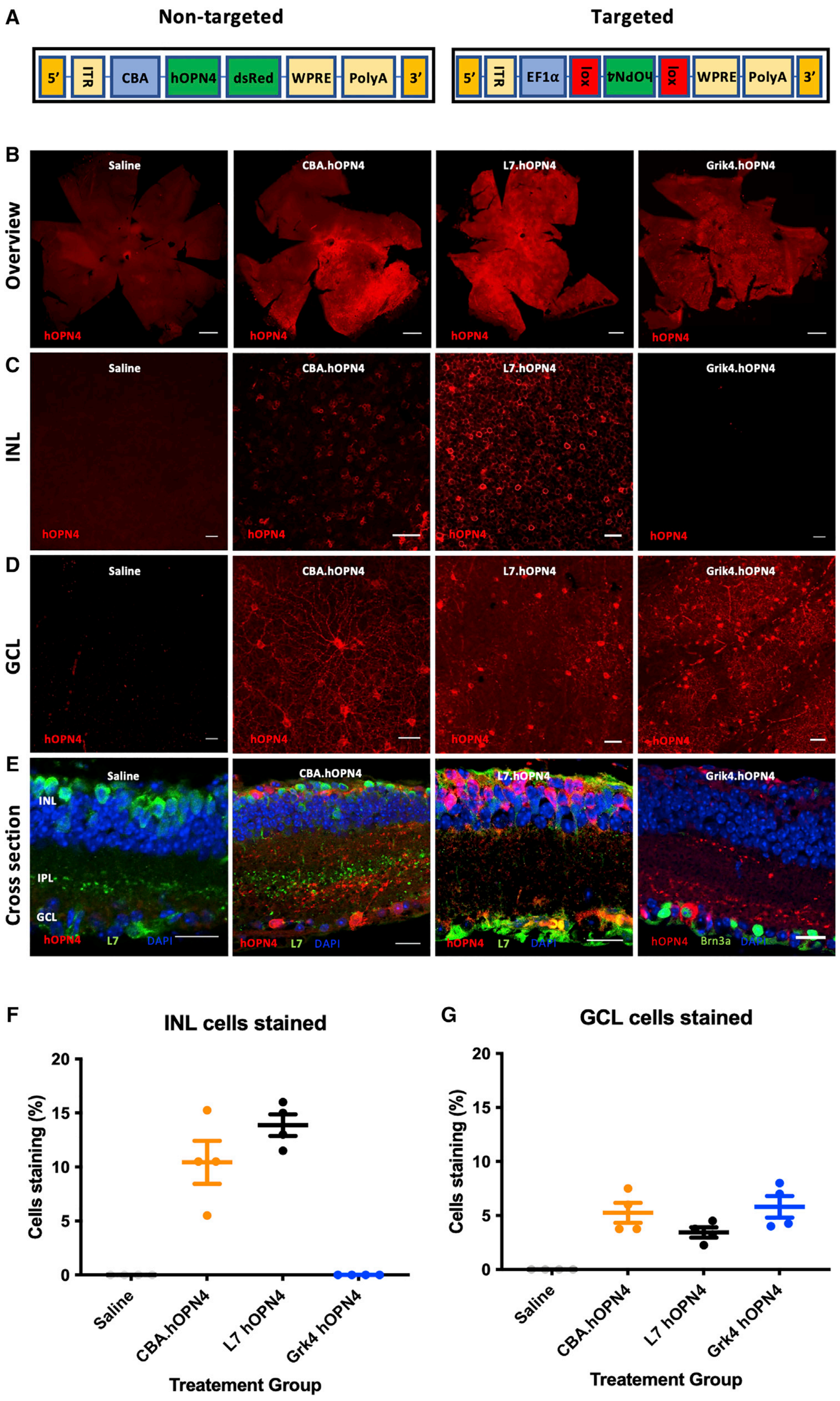

(legend on next page)

tool will function, and it is this combination that will ultimately define suitability for optogenetic therapy.

To date, only a very small number of studies have directly compared candidate optogenetic tools, and none have systematically addressed the role of the different classes of optogenetic tools and target cell populations in the same model system.[3,5–8,20–23] In addition, none have compared responses in a degenerate model devoid of both rod, cone, and melanopsin responses[7,23,24] to remove the confounder of residual native light responses.

With this small number of directly comparative studies, many of the relative advantages and disadvantages of particular optogenetic tools remain somewhat theoretical in relation to IRDs. The aim of this study is to directly compare the sensitivity and kinetics of two leading tools, namely human melanopsin (hOPN4)[6,25,26] and the red-shifted channelrhodopsin ReaChR,[10] when expressed either non-specifically or within relatively defined subpopulations of surviving cells in the degenerate retina. These two tools were chosen as reasonably well-characterized examples (at both the physiological and behavioral levels) of the two main classes of optogenetic tools: mammalian opsins (hOPN4) and microbial ion channels (ReaChR). The promoter constructs (chicken beta actin [CBA; non-specific], L7 [ON bipolar cell dominant], Grik4 [retinal ganglion cell dominant]) were chosen as examples where the cell populations they target are well described in various contexts. As both comparisons indicated a functional advantage to targeting populations rich in upstream neurons, we proceeded to develop a clinically translatable promoter targeted gene delivery strategy enabling optogenetic tool expression in the ON bipolar cell population.

RESULTS

Comparing sensitivity and kinetics of hOPN4 when targeted non-specifically and to L7 and Grik4 subpopulations of retinal cells as an optogenetic tool

Restricting expression to defined subpopulations using the Cre/lox system

Ongoing clinical trials employ a transduction strategy to broadly express optogenetic tools in multiple surviving neuronal cell types (ClinicalTrials.gov: NCT04945772, NCT02556736 NCT04919473, and NCT03326336; clinicaltrials.gov). To replicate this, we employed AAV (AAV2/2 quad mutant Y272,444,500,730F) to deliver hOPN4 driven by a high efficiency, non-specific promoter (CBA) via intravitreal injection (Figure 1A). Consistent with previous reports,[6] this achieved expression of hOPN4 protein throughout the mouse retina, with preferential expression in horizontal cells and retinal ganglion cells (Figures 1B–1G), hereafter referred to as CBA.hOPN4. In order to directly compare this non-specific delivery, a modified AAV construct incorporating a floxed hOPN4 gene (flox.hOPN4) (Figure 1A) was used to restrict expression only to cells expressing the Cre recombinase enzyme in our transgenic mouse models.

When flox.hOPN4 was administered to the Grik4.Cre mouse (Grik4.hOPN4), protein staining was seen in a subset of retinal ganglion cells (RGCs) as previously described.[27] When the flox.hOPN4 virus was administered to the L7.cre mice (L7.hOPN4), staining was seen within a proportion of L7-expressing bipolar cells[28] of the inner nuclear layer (Figures 1C and 1F) together with a small subpopulation of RGCs, as previously described[27] (Figures 1D and 1G).

Targeting hOPN4 to defined subpopulations of cells evokes distinct response characteristics

Eight weeks after intravitreal injection, CBA.hOPN4, L7.hOPN4, and Grik4.hOPN4 mice were culled for *ex vivo* multiple-electrode array (MEA) electrophysiological recordings. In all three treatment groups, exogenously expressed hOPN4 protein was able to drive changes in spike firing of RGCs in response to light; there were no such changes in saline control groups, confirming the absence of native melanopsin responses (Figures 2A and 2B). While hOPN4-treated groups had significantly more light-responsive electrodes than saline groups (one way ANOVA, $F_{(3,21)} = 4.136$; $p < 0.0188$ with Tukey's post hoc test), there were no significant differences in the proportion of light-responsive electrodes between treatment groups (Figure 2C).

The half-life ($t_{1/2}$) of light responses provides an overarching index of decay kinetics in each group. This value lengthened with stimulus intensity ($F_{(6,2116)} = 137.2$; $p < 0.0001$) and varied between treatment groups ($F_{(2,2116)} = 29.68$; $p < 0.0001$). This suggests that while brighter stimuli led to generally longer $t_{1/2}$, decay kinetics were significantly shortened when hOPN4 was targeted to L7-positive ON bipolar cells compared with RGCs or non-specific delivery (see Figure 2D for post hoc test).

Irradiance response curves (IRCs; Figure 2E) revealed that there was no significant difference in the half maximal effective concentration (EC_{50}) between CBA.hOPN4 (13.74 ± 0.11 $\log_{10}$ photons cm^{-2} s^{-1}) and L7.hOPN4 (13.64 ± 0.21 $\log_{10}$ photons cm^{-2} s^{-1}) (one-way ANOVA $F_{(2,109)} = 18.31$; $p < 0.0001$; Tukey's post hoc test; $p = 0.8053$), while

Figure 1. Intravitreal delivery of hOPN4 using adeno-associated viral vectors

(A) Linearized representation of insert plasmids used to make non-targeted (CBA.hOPN4) and targeted (flox.hOPN4) vectors. Inverted sequences are shown upside down. (B) IHC of retinas 4 weeks post intravitreal injection with the indicated AAV/mouse line. Scale bar, 500 μm. (C and D) From retinas in (B), focused on the layer of the inner nuclear layer (C) or the ganglion cell layer (D). Scale bar, 20 μm. (E) Retinal cross-section IHC counterstained for L7 or Brn3a. Scale bar, 20 μm. (F) Proportion of cells staining for hOPN4 in the INL. Each point is the mean value taken over four 40× fields of view (n = 4) from one animal (N = 4). One-way ANOVA $F_{(2,9)} = 31.37$, $p < 0.0001$; Tukey's post hoc test: no significant difference between CBA.hOPN4 and L7.hOPN4, $p = 0.1977$. (G) As in (F), but for GCL. No significant difference between treatment groups. One-way ANOVA $F_{(2,9)} = 2.243$, $p = 0.1620$. Unless otherwise indicated, data is presented as mean±standard error of the mean. ITR, inverted terminal repeat; WPRE, woodchuck hepatitis virus post transcriptional regulatory element; PolyA, Poly(A) tail; EF1α, elongation factor 1 alpha; CBA, chicken beta actin; hOPN4, human melanopsin; INL, inner nuclear layer; IPL, inner plexiform layer; GCL, ganglion cell layer; IHC, immunohistochemistry.

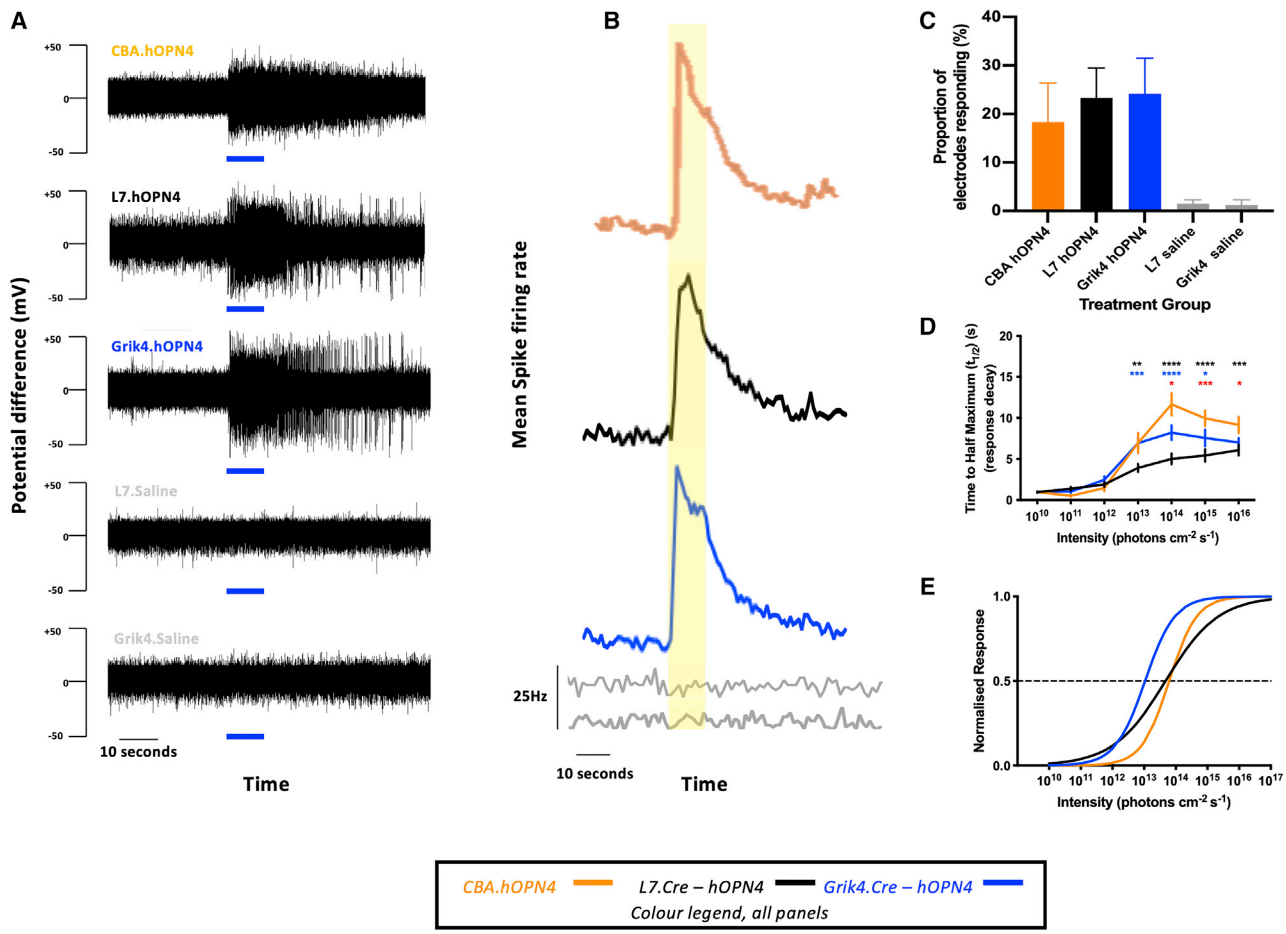

Figure 2. Multiple-electrode array (MEA) electrophysiology – hOPN4

(A) Example raw recording traces from a single electrode in each treatment group stimulated with a 10 s pulse of 480 nm, 10^{14} photons cm^{-2} s^{-1} light (blue bar). (B) Mean change in spike firing rate from all responsive electrodes in each group in response to a 10 s pulse of 480 nm, 10^{14} photons cm^{-2} s^{-1} light (yellow shading). (C) The proportion of electrodes in each group scored as responsive. No significant difference between treatment groups (see text, Supplemental material, and Table S5. (D) Response decay kinetics: the time taken from maximum response to half maximum response ($t_{1/2}$). Stars refer to post hoc test (Tukey's) following two-way ANOVA (see text). Black stars refer to CBA.hOPN4 versus L7.hOPN4; blue stars to L7.hOPN4 versus Grik4.hOPN4; red stars to Grik4.hOPN4 versus CBA.hOPN4. *$p < 0.05$, **$p < 0.01$, ***$p < 0.001$, ****$p < 0.0001$. (E) Irradiance responses curves (IRCs) were plotted using the mean EC_{50}, and Hill slope values were derived from averaging those of individual electrode fits. Electrodes with fits $r^2 < 0.8$ were excluded from analysis. See text and Figures 3G and 3K for details of statistical comparisons. CBA.hOPN4: EC_{50} = 13.74 ± 0.11 photons cm^{-2} s^{-1}; Hill slope = 0.8831 ± 0.06, r^2 = 0.9348 ± 0.0096, n = 35 electrodes; L7.hOPN4: r^2 = 0.9061 ± 0.0106, n = 20; Grik4.hOPN4: r^2 = 0.9426 ± 0.0061, n = 57. Unless otherwise indicated, data is presented as mean±standard error of the mean.

Grik4.hOPN4 (13.03 ± 0.06 log_{10} photons cm^{-2} s^{-1}) was significantly more sensitive than hOPN4 delivered either non-specifically or to L7 ON bipolar cells (Tukey's post hoc test; $p < 0.0005$ for both comparisons). The Hill slope of a sigmoidal IRC provides a measure of the range of light intensities the modeled opsin is able to encode (i.e., its dynamic range). With L7.hOPN4, the Hill slope was significantly lower than for hOPN4 delivered non-specifically or targeted to RGCs (one-way ANOVA $F_{(2,109)} = 6.853$; $p < 0.0001$; Tukey's post hoc test; $p < 0.01$ for both comparisons). The dynamic range of hOPN4 is approximately two orders of magnitude when expressed in RGCs or non-specifically but extends over five orders of magnitude when expressed in L7-positive ON bipolar cells, a very significant difference when considering clinical translation. Both this advantage in dynamic range with L7-positive bipolar cell targeting and in absolute sensitivity with Grik4-positive RGC targeting could be explained by differences either in the signaling cascade dynamics within the transduced cells or their connections to other cells (e.g., the retention of an additional synaptic layer with L7-positive bipolar targeting allowing integration of overlapping individual bipolar cell ranges).

Comparing targeted delivery of receptor- and channel-based optogenetic tools

We next sought to investigate if these differences in kinetics and sensitivity of response were dependant on the tool used or if they were attributable to features intrinsic to the targeted cell population (and therefore expandable to distinct classes of optogenetic tools). We

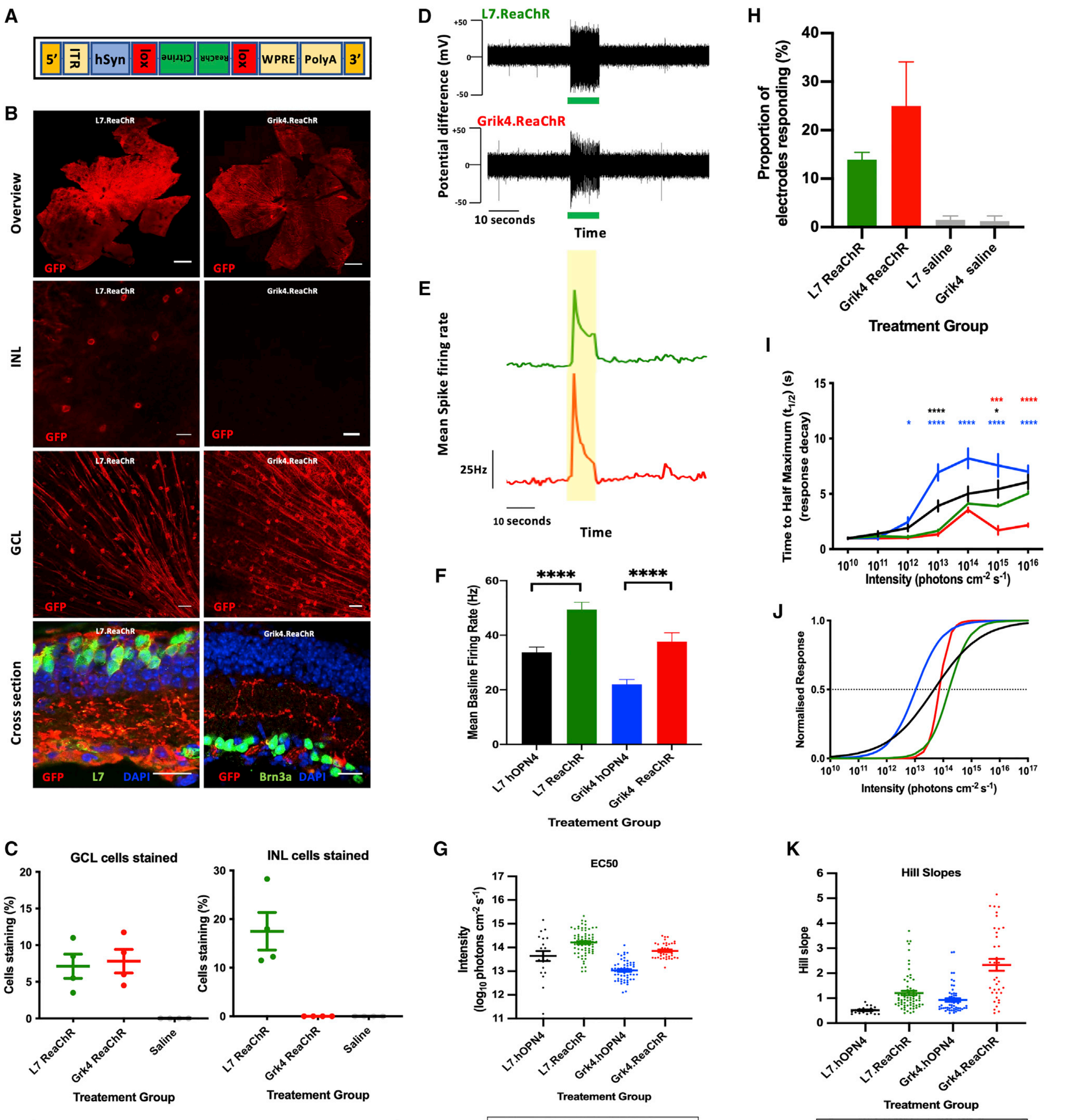

Dunn's multiple comparison test, p= (G)

	L7.hOPN4	L7.ReaChR	Grk4.hOPN4	Grk4.ReaChR
L7.hOPN4		0.021	0.0013	>0.9999
L7.ReaChR	0.021		<0.0001	0.0924
Grk4.hOPN4	0.0013	<0.0001		<0.0001
Grk4.ReaChR	>0.9999	0.0924	<0.0001	

Dunn's multiple comparison test, p= (K)

	L7.hOPN4	L7.ReaChR	Grk4.hOPN4	Grk4.ReaChR
L7.hOPN4		<0.0002	0.0028	<0.0001
L7.ReaChR	<0.0002		0.0634	0.0043
Grk4.hOPN4	0.0028	0.0634		<0.0001
Grk4.ReaChR	<0.0001	0.0043	<0.0001	

Figure 3. Targeted hOPN4 and ReaChR delivery

(A) Insert plasmid used to make flox.ReaChR vector. Inverted sequences are shown upside down. (B) IHC showing expression of ReaChR.mCitrine. Please note that GFP antibody cross-reacts with mCitrine in flox.ReaChR construct, hence its use here. An additional saline control (not shown) was stained for GFP with appearances similar to that stained for hOPN4 in Figure 1. Scale bars: whole retina, 500 μm; otherwise, 20 μm. (C) Proportion of all cells staining for GFP in the INL and GCL. Each point is the mean value taken over four 40× fields of view (n = 4) from one animal (N = 4). GCL: one-way ANOVA $F_{(1.297,3.890)} = 19.24$, $p < 0.0113$; Tukey's post hoc test: L7.ReaChR versus

(legend continued on next page)

addressed this question by repeating the same set of experiments using a structurally and functionally unrelated optogenetic tool as a control. ReaChR[10] is a photoactivated ion channel and so, unlike hOPN4 (a G-protein coupled receptor), does not couple to an amplifying second-messenger cascade and thus is expected to produce less sensitive responses but with faster kinetics.

Delivery of a floxed ReaChR construct (Figure 3A) resulted in cell-specific expression of ReaChR protein in both L7.Cre and Grik4.Cre mice (Figures 3B and 3C). As with hOPN4, expression of ReaChR was able to drive light responses (Figures 3D and 3E). However, the mean baseline (10 s pre-stimulus) firing rates varied significantly between groups (one-way ANOVA $F_{(3,334)} = 13.23$; $p < 0.0001$), with significantly higher rates in both ReaChR-transfected groups compared with hOPN4 (Figure 3F). Treated groups had significantly more light-responsive electrodes than saline controls (one-way ANOVA, $F_{(3,28)} = 11.61$; $p < 0.0001$), but there was no difference in the proportion of light-responsive electrodes between the two ReaChR groups (Tukey's post hoc test; $p = 0.0943$).

Total response $t_{1/2}$ in ReaChR groups increased with increasing intensity (two-way ANOVA $F_{(63,407)} = 14.81$; $p < 0.0001$) and varied with treatment group ($F_{(33,407)} = 107.1$; $p < 0.0001$). Again, as expected,[6,10,20] brighter stimuli led to longer $t_{1/2,}$ but decay kinetics were faster in ReaChR compared with hOPN4-treated groups, especially in the Grik4.ReaChR group at higher intensities (Figure 3I). Spike adaptation (measured as the $t_{1/2}$ of responses from the point of maximum spike firing to that of stimulus offset) was not significantly different between groups (Kruskal-Wallis [KW] test, KW statistic = 3.181, $p = 0.3646$). Full offset kinetics (the $t_{1/2}$ of responses from the point of response offset) varied between groups (KW test, KW statistic = 38.07, $p < 0.0001$); however, Dunn's post hoc test was only significant for comparisons between tools, not target cell populations (L7.hOPN4: 11.82 ± 1.84 s; L7.ReaChR: 3.143 ± 0.50 s; Grik4.hOPN4: 10.43 ± 0.99 s; Grik4.ReaChR: 3.86 ± 0.83 s).

Again, a sigmoidal irradiance response relationship could be observed for both conditions (Figure 3J). There was no significant difference in EC_{50} between ReaChR groups, regardless of where the tool was targeted; ReaChR groups were less sensitive to light than hOPN4 when targeted to the same populations (KW test with Dunn's post hoc test; KW statistic = 93.46, $p < 0.0001$; see Figure 3G for post hoc test values). Similarly, there were significant differences in the Hill slope between groups with a lower value when treatment was targeted to L7-positive ON bipolar cells as opposed to Grik4-positive RGCs, similar to that seen for hOPN4 (KW test with Dunn's post hoc test; KW statistic = 61.93, $p < 0.0001$; see Figure 3K for post hoc values).

A compressed L7 promoter construct can be used to selectively express functional hOPN4 in ON bipolar cells, without the need for the Cre/lox system

There is an increasing body of evidence to suggest that intraretinal signal processing can be maintained by targeting surviving upstream retinal neurons, especially bipolar cells.[13,17–19,29] The flatter stimulus intensity-response relationship and shorter decay time further support ON bipolar cells as favorable targets for optogenetic vision restoration. Despite recent advances,[30] obtaining such targeted expression within bipolar cells remains challenging. The large size of the L7 promoter (2.9 kbp) precludes its integration with an optogenetic tool within the c.5 kbp packaging capacity of an AAV.[31] Recently, a compressed L7 promoter (L7-6) has been developed and has proven efficient in both mice and primates.[32] We hypothesized that such a promoter could be a valuable and clinically translatable tool to obtain specific expression of hOPN4 within L7-positive ON bipolar cells.

In a series of pilot experiments, we therefore constructed AAV carrying hOPN4 under the control of the L7-6 construct (L7-6.hOPN4; Figure 4A). Using immunocytochemistry, we confirmed that this vector was able to drive the production of human hOPN4 protein in L7-expressing bipolar cells in primary murine retinal cell culture. Based on these *in vitro* culture findings, we proceeded to intravitreally inject L7-6.hOPN4 into the eyes of 4-week-old retinal degenerate, melanopsin-deficient (*Pde6b*$^{rd/rd1}$ *Opn4*$^{tm1yau/tm1yau}$) mice. Four weeks later, retinal tissue was collected for immunohistochemistry (IHC) showing staining for hOPN4 in cells located in the inner nuclear layer (INL) that also co-stained for L7 protein. A small number of cells in the ganglion cell layer (GCL) were stained, consistent with findings in the L7 Cre.lox model.[27] Additionally, we confirmed that this approach results in the expression of functional hOPN4 and is able to drive changes in the spike firing rate of RGCs in responses to light (Figures 4B–4I). This light responsiveness was seen in a similar proportion of electrodes (Figure 4G) to that seen

Grik4.ReaChR, $p = 0.6469$; ICL: one-way ANOVA: $F_{(1,3)} = 20.49$, $p = 0.0202$; Tukey's post hoc test: L7.ReaChR versus Grik4.ReaChR, $p = 0.0406$. (D) Example raw recording traces from a single electrode in each treatment group stimulated with a 10 s pulse of 510 nm, 10^{14} photons cm^{-2} s^{-1} light (green bar). (E) Mean change in spike firing rate from all responsive electrodes in each group in response to a 10 s pulse of 510 nm, 10^{14} photons cm^{-2} s^{-1} light (yellow shading). (F) The mean baseline firing rate in the 10 s preceding the 10^{15} photons cm^{-2} s^{-1} in each group. (L7.hOPN4 and Grik4.hOPN4 data redrawn from Figure 2C for context). (G) A Kruskal-Wallis test (see text) with Dunn's multiple comparison test was used to compare the mean EC_{50} (G) and Hill slope (K) values. (H) The proportion of electrodes in each group scored as responsive. No significant difference between treatment groups (see text). (I) Response decay kinetics: the time taken from maximum response to $t_{1/2}$. Red asterisks refer to comparisons between ReaChR groups (Tukey's post hoc test), black to comparisons between L7.Cre groups, and blue to comparisons between Grik4.Cre groups. (L7.hOPN4 and Grik4.hOPN4 data redrawn from Figure 2D for context). (J) Irradiance responses curves (IRCs) were plotted using the mean EC_{50} and Hill slope values derived from averaging those of individual electrode fits. Electrodes with fits $r^2 < 0.8$ were excluded from analysis. L7.ReaChR: $r^2 = 0.9443 \pm 0.001$, n = 71; Grik4.ReaChR: $r^2 = 0.9342 \pm 0.007$, n = 36 (L7.hOPN4 and Grik4.hOPN4 data redrawn from Figure 2E for context). ITR, inverted terminal repeat; WPRE, Woodchuck hepatitis virus post transcriptional regulatory element; PolyA, Poly(A) tail; EF1α, elongation factor 1α; CBA, chicken beta actin; hOPN4, human melanopsin. Promoters are in blue. *p < 0.05; **p < 0.01, ***p < 0.001, ****p < 0.0001. Unless otherwise indicated, data is presented as mean±standard error of the mean.

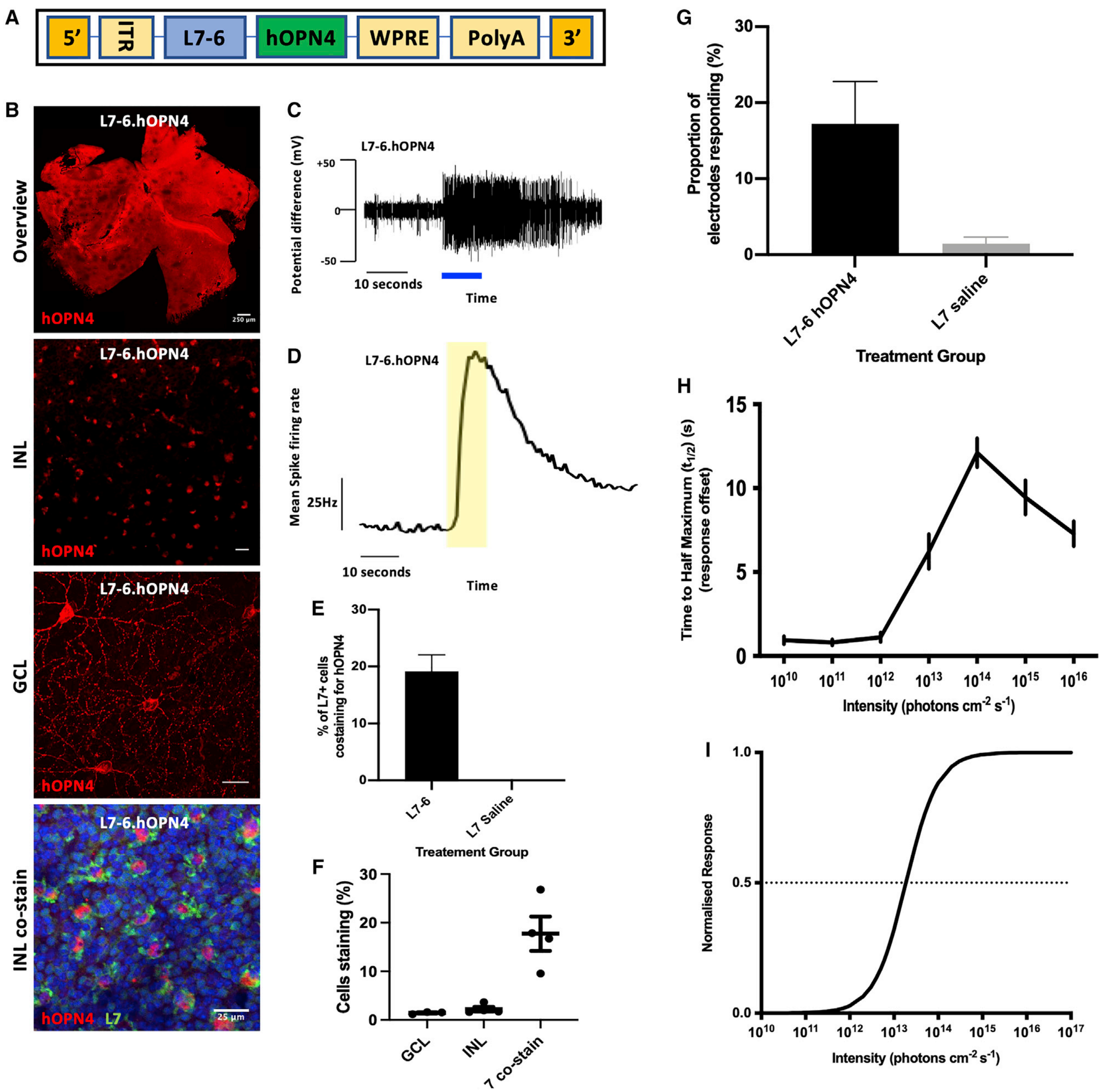

Figure 4. hOPN4 delivered using the L7-6 promoter

(A) Insert plasmid used to make L7-6 hOPN4 vector. (B) IHC showing expression of L7-6 hOPN4. Scale bars: whole retina 250 μm; otherwise, 25 μm. (C) Example raw recording traces from a single electrode in each treatment group stimulated with a 10 s pulse of 480 nm, 10^{14} photons cm^{-2} s^{-1} light (blue bar). (D) Mean change in spike firing rate from all responsive electrodes in each group in response to a 10 s pulse of 480 nm, 10^{14} photons cm^{-2} s^{-1} light (yellow shading). (E) Proportion of L7-expressing cultured primary dissociated mouse retina cells incubated with L7-6 hOPN4 AAV that also express Opn4. Treatment groups, n = 197 total L7 cells; saline, n = 213 cells (unpaired t test $p < 0.0010$). (F) Whole retina IHC: cells staining for hOPN4 in the GCL and INL (as a proportion of all cells) compared with the proportion of cells staining for hOPN4 as well as L7 in the INL. N = 4 retinas each. (G) The proportion of electrodes scored as responsive. N = 4 retinas, n = 32 electrodes. (unpaired t test $p < 0.0010$). (H) Response decay kinetics: the time taken from maximum response to $t_{1/2}$. (I) IRCs were plotted using the mean EC_{50}, and Hill slope values were derived from averaging those of individual electrode fits. L7-6 hOPN4: EC_{50} 13.27 ± 0.09 photons cm^{-2} s^{-1}; Hill slope 1.21 ± 0.11, r^2 = 0.9380 ± 0.009, n = 31. GCL, ganglion cell layer; INL, inner nuclear layer; ITR, inverted terminal repeat; L7-6, L7-6 promoter; hOPN4, human melanopsin gene; WPRE, Woodchuck hepatitis virus post transcriptional regulatory element; PolyA, Poly(A) tail. Unless otherwise indicated, data is presented as mean±standard error of the mean.

with Cre.lox-mediated delivery, with a peak $t_{1/2}$ around 10^{14} photons cm^{-2} s^{-1} (Figure 4H), and fitted a sigmoidal irradiance response relationship with an EC_{50} of 13.27 ± 0.09 photons cm^{-2} s^{-1} (Figure 4I).

DISCUSSION

In this study, we describe a direct comparison of the optogenetic tool hOPN4 expressed non-specifically and within defined subpopulations of the degenerate retina. We show that targeting different cell groups markedly alters the reconstituted light responses. Furthermore, we demonstrate the use of the compressed L7-6 promoter construct to deliver a gene (hOPN4) specifically to a population rich in retinal ON bipolar cells, a technique that could potentially allow direct clinical translation of this work.

Subpopulation targeting of hOPN4 produces differing response characteristics

While the wider dynamic range seen when either tool was targeted to L7-positive ON bipolar cells is potentially useful for clinical translation of the technique, it may have been expected given the additional synaptic layer retained allowing the integration of overlapping ranges of upstream bipolar cells. However, the markedly increased absolute sensitivity seen when RGCs were targeted with melanopsin could also have utility in providing perception in lower-light conditions. Several factors could contribute to these differences in sensitivity and dynamic range including the network interactions and intrinsic neurophysiological properties of this cell population, e.g., lower membrane resting potential, and these may change depending on the exact capsid, promoter construct, and tool employed.

However, in our comparison, the observation that increased sensitivity of hOPN4 was not replicated when ReaChR was similarly targeted may also be explained by melanopsin coupling to an alternative second-messenger system in RGCs compared with ON bipolar cells. Melanopsin is reported to couple to different G protein systems *in vitro*[33,34] and *in vivo*[35–37] depending on the cellular environment in which it is expressed. As a $G_{q/11}$-coupled G protein, natively expressed in a subtype of RGC, melanopsin may indeed find a preferred cascade to couple to when expressed in RGCs, increasing sensitivity when compared with bipolar cells where $G_{q/11}$ signaling is less prominent. Differential coupling may also be important in explaining the expediated decay kinetics seen when melanopsin is expressed in the L7-positive ON bipolar cell population. In its native cell (the intrinsically photosensitive RGC), hOPN4 has been proposed to have its response terminated by activity-dependent phosphorylation of a region of its C terminus,[38–42] which allows arrestin-β2[43–45] to bind. The presence of cell-specific kinases in L7-positive bipolar cells, e.g., protein kinase C alpha (PKCα),[46] central to the termination the ON bipolar native light response,[47–51] may represent a potential mechanism if PKCα were able to facilitate this phosphorylation of melanopsin. Indeed, further investigation of hOPN4 coupling and response termination in ON bipolar cells could provide a fruitful avenue of investigation to further optimize its sensitivity and kinetics for therapeutic use.

Comparing targeted delivery of two candidate optogenetic tools

Baseline firing

Key differences between mammalian and microbial opsins, in particular sensitivity and kinetic behaviors, have been extensively discussed.[14,29] Yet, the functional differences we observed comparing these tools in the context of our comparison extended beyond this: in particular, the higher baseline firing rates seen in all ReaChR groups compared with hOPN4. Changes in baseline firing have been noted in optogenetically treated rd1 retina previously.[52] Indeed, *in vitro* studies have suggested a small amount of leak current is present with exogenously expressed channelrhodopsins, allowing ingress of cations (notably Ca^{2+}) with repeated activation.[53,54] This could presumably lead to a slight, relative depolarization of the membrane potential, so accounting for both an increased spontaneous baseline and higher post stimulus spike firing rate in these groups. The potential long-term toxicity of a leak current to retinal neurons specifically has not been investigated, but there are suggestions of channelrhodopsins causing toxicity in other tissues.[55,56] While more detailed characterization of increased baseline firing is indicated as these tools move toward the clinic, in this study, it does not appear to impair cells' ability to respond to light stimuli with spike trains or, indeed, significantly alter the proportion of responsive electrodes.

Sensitivity and kinetics

Indeed, in the move toward translation, the lower sensitivity of microbial tools (such as ChannelRhodopsin2 [ChR2][57]) has been cited as a disadvantage, with extraocular amplification equipment often required.[58] While the absolute levels of sensitivity with ReaChR seen here are higher with targeted delivery, in this study, they are not yet comparable to hOPN4 deployed to the same cell population. Additionally, ReaChR groups demonstrated narrower dynamic ranges than hOPN4 ones, likely a combination of both high open probabilities of individual channels[58] and the lack of second-messenger coupling. Indeed, in terms of sensitivity and dynamic range, hOPN4 would appear to be the more attractive tool in both populations investigated here and compares favorably with other opsin tools on these metrics (when used for visual restoration, cone or rhodopsin required similar or even higher stimulus intensities).[3,5,59]

However, the lower sensitivity of ReaChR is countered by the markedly expediated decay kinetics when it was targeted to either cell population in this study. This is additionally interesting in the context of previous observations that ReaChR is better able to track sinusoidal stimuli when targeted to an L7-positive bipolar population, compared with non-specific delivery.[13] These findings would imply a better temporal resolution and would warrant further development, given the need for such resolution for the restoration of naturalistic vision.

Limitations

In developing potential cell-population-specific optogenetic vision-restoration therapies, however, care must be taken when extrapolating findings as levels of protein expression in individual cells can vary depending on not only the capsid and promoter used but also

the species and specific state of the retina,[60,61] as well as the route of delivery and concentration of vector. Additionally, few promoter constructs are completely specific for a sole type of retinal cell.

Considering these limitations, we selected two Cre promoter constructs with relatively well-described off-target expression, integrated high-efficiency promoters (E1α and hSyn) into the AAVs, and injected "neat" (undiluted) vectors intravitreally to maximize expression. While this approach was chosen to maximize expression, we cannot exclude that our observations in this study may be partially biased by different transduction efficiencies caused by using different viral titers and promoters between targeted groups.

The Grik4 construct is known to restrict expression to a well-defined population of direction-sensitive RGCs[28] and L7 mainly to retinal ON bipolar cells[27,28] in mice (such as the widely used rd1 model of retinal degeneration employed here). However, in common with other bipolar-cell-targeting constructs (e.g., grm6-SV40),[5,8,12,17,18,62,63] L7 has some off-target expression.[27,28] For ease, throughout this report, these Grik4 and L7 populations have been referred to as RGCs and ON bipolar cells; however, this off-target expression must be considered when extrapolating conclusions to other situations.

Patterns of off-target expression and variable levels of protein expression between cells will be inherent to any combination of promoter and capsid. T -his will persist through to translation here we selected well-described transgenes, promoters, capsids, and models. This allowed us to represent the most relevant classes of tool and target populations - incorporating off-target effects and variability in quantity of expression to give a useful functional comparison for the first time. This will help to direct the future detailed comparisons required to replicate our findings with other promoter-capsid combinations and isolate individual cell responses.

Compressed promoter delivery of hOPN4

The targeting of retinal ON bipolar cells with specific promoters has several advantages for functional optogenetic vision restoration—not least the retention of an additional intraretinal synapse to retinal image processing. Moreover, we have recently shown that these cells undergo relatively little transcriptomic changes in the context of IRDs, making them an attractive target for optogenetic gene therapies despite retinal remodeling.[64–72]

These advantages, together with the increased dynamic range and favorable response kinetics we observed when hOPN4 was targeted to the L7-positive bipolar cell population, led us to combine hOPN4 with a repurposed compressed (shortened) L7-6 promoter construct.[32] One of the barriers to population-targeted gene therapies is the limitations in payload size of AAV vectors: many specific promoters (including L7) are too large to fit along with a transgene within the vector envelope. The L7-6.hOPN4 construct, delivered by AAV vector, was able to target functional hOPN4 to L7-positive bipolar cells in a degenerate retina without the need for the Cre/lox system. Indeed, further work is required to expand this preliminary investigation—for example, by using L7-6 to deliver alternative tools such as ReaChR and to characterize the restored light responses more fully. However, by providing an alternative method to deploy cell-population-specific optogenetics via proven AAV vectors, this represents a step toward clinical translation for cell-targeted melanopsin optogenetics. Given the demonstrated differences in other promoters specificity in degenerate retinas[61] and when moving to primates,[60] L7-6 appears to be particularly attractive and adds to a growing armory of retinal-cell-population-specific mini-promoters.[30,73–77]

Comparing optogenetic targets and tools: Toward therapeutic optogenetics in IRDs

An overarching aim of this study was to functionally compare approaches to optogenetics in a retinal degeneration model, ultimately to help determine which tool, delivered by which approach, would best facilitate restoring naturalistic physiological responses to light. At the most basic level, such responses would ideally be large in amplitude, have fast kinetics, and be sensitive over a wide dynamic range. From the investigations described above, it could be concluded that there was no one combination that would meet all these criteria in this context. The remarkable diversity of electrophysiological responses to the same stimuli produced by targeting different optogenetic tools to different populations of retinal cells was particularly notable. For example, the diversity of spike firing responses demonstrated in Figures 2A and 3D are evocative of the diversity of native RGC-subtype responses (e.g., L7.ReaChR to sustained ON-type RGCs).[78]

As targeted optogenetics develops, the diversity of responses could indeed be a great advantage. An increased understanding of response characteristics of specific tools in specific cell populations could allow a calculated, multi-tool, multi-target approach. It could be envisioned that several tools, targeted to different retinal cell types, could replicate separate aspects of the wild-type retina's diverse RGC spike firing responses or indeed a diversity of responses that plastic higher visual centers could learn to make use of, both of which could ultimately lead to a more naturalistic restored visual experience for the IRD patient.

CONCLUSION

Here, we describe the first direct comparison of optogenetic tools targeted to different subpopulations of surviving cells in a degenerate retina. The diversity of responses observed, and the clinically translatable delivery strategy described, hold great potential to improve the quality of optogenetic vision-restoration strategies for patients with IRDs.

MATERIALS AND METHODS

Mice

All experiments involving animals were performed in accordance with the Animals for Scientific Procedures Act 1986, licence numbers 30/3371 and PE4ED9D2C, and the University of Oxford policy on the use of animals in scientific research and in accordance with the ARVO Statement for the Use of Animals in Ophthalmic and Vision

Research. Animals were kept under a 12:12 h light/dark cycle, with food and water *ad libitum*.

Two transgenic lines were bred from predecessor lines (see Supplemental methods and Table S1) to produce mice homozygous for both the retinal degeneration causing mutation *Pde6b*^{rd1}[79] and the knockout *Opn4*^{tm1yau} allele[80] while additionally expressing the Cre recombinase enzyme under the control of the ON-bipolar-cell-specific L7 promoter (alias *Pcp2*)[81,82] or ganglion-cell-specific promoter *Grik4*[28,83] (see Supplemental methods and Table S2). The expected melanopsin-deficient, retinal-degenerate retinal phenotype was confirmed by IHC (Supplemental methods; Tables S3 and S4).

AAV vectors

Three AAV vectors were produced following previously described methods[13,84] to incorporate the hOPN4 and ReaChR constructs illustrated in Figures 1A, 1B, and 3A.[6,85] In brief, HEK293T cells were co-transfected with these constructs, along with capsid (AAV2/2 quad mutant Y272,444,500,730F) and helper plasmids before cell lysis and purification using an iodixanol gradient and subsequent centrifugal concentration.[13,84] Final purified virus was suspended in physiological concentration saline. Retinal expression of the expected proteins was confirmed by IHC (Supplemental methods).

L7-6 compressed promoter construct

A compressed promoter construct L7-6,[32] which has proven efficient at inducing cell-type-specific gene expression in cerebellar granule neurons but hitherto not examined in the retina, was kindly received from Prof. Hirai (Gunma University, Japan) and cloned into an AAV insert backbone along with the hOPN4 gene (Figure 4A) using an MluI/KpnI restriction enzyme approach. The resulting plasmid was used to produce AAV and was validated as described above.

Intraocular injections

Bilateral intravitreal injections were administered to mice under isoflurane anesthesia at 6 to 8 weeks of age following previously published methods[13,84] (Supplemental methods). L7.Cre mice were injected with each of the three AAVs (Figures 1A and 3A) or saline (eight mice per group). Grik4.Cre mice were injected with the two floxed viruses (flanked by *LoxP*, i.e., requiring Cre recombinase within the target cell to be expressed) (Figures 1A and 3A) or saline (eight per group). Eight *Pde6b*$^{rd/rd1}$ *Opn4*$^{tm1yau/tm1yau}$ tg(L7.Cre)$^{WT/WT}$ mice were injected with L7-6.hOPN4 AAV (Figure 4A).

IHC

IHC was performed following established in-house protocols[13,84]. Briefly, directly after enucleation at 4–6 weeks post injection, eyes were transferred to 4% paraformaldehyde (PFA; Alfa Aesar, Ward Hill, MA, USA) for 24 h before cryoprotection with 30% sucrose-PBS for 48 h. Eyes were embedded in optimal cutting temperature (OCT) solution (Finetek, Torrance, CA, USA), and 20 μm sections were placed onto poly-lysine-coated slides (VWR, Leuven, Belgium). Permeabilisation with 0.2% Triton X–PBS (Sigma Aldrich, Gillingham, UK) was followed by blocking with 0.2% Triton X–PBS incorporating 10% serum from the animal in which the secondary antibody was raised (Table S4). Overnight primary antibody incubation was in 0.2% Triton-PBS X with 2.5% serum, followed by three 10 min washes in 0.1% Tween-PBS and overnight incubation with the secondary antibody at a concentration of 1:250 in 0.2% Triton X–PBS with 2.5% serum. Nuclei were stained with 300 nmol/L 4′,6-Diamidino-2-Phenylindole (DAPI) (Thermo Fisher, Loughborough, UK) before mounting with ProLong Diamond mounting media (Thermo Fisher, Loughborough, UK). Retinal sections were visualized using a confocal microscope (LSM710; Zeiss, Oberkochen, Germany), and images were processed using Fiji-ImageJ.[86] A similar method was used to stain flat mount retinas and is included in the supplemental methods. For each animal, four non-continuous 40× fields of view were selected from the flat mount as subjectively showing the highest levels of staining, and for each of these, the number of cells staining for hOPN4/GFP and the number only staining for DAPI were counted to give a proportion of stained cells.

MEA recordings

Eight weeks following intraocular injections, mice were culled by cervical dislocation under dim red light (>610 nm). Dissected retinas were immediately transferred to glass-bottomed MEA chambers (Multi Channel Systems, Reutlingen, Germany) with a grid of 60, and 30 μm recording electrodes were spaced 200 μm apart and continuously perfused with Ames' medium (Sigma Aldrich, Gillingham, UK) bubbled with 5% CO_2 (pH 7.3) at 33°C. Signals were amplified, digitized (at 25 kHz), recorded using the MC Rack software suite (Multichannel Systems, Reutlingen, Germany), and analyzed using a custom MS Excel spreadsheet (Supplemental materials). Retinas were equilibrated in the dark for 40 min before onset of experimental recordings (see Supplemental methods).

Light stimuli were produced by an X-cite 120W metal halide light source (Excelitas, Southampton, UK) and conditioned by passing through spectral filters (480 ± 10 nm for hOPN4 groups or 510 ± 10 nm for ReaChR groups, corresponding to their respective λ_{max}) and neutral density filters to give one of seven intensities of light (10^{10} to 10^{16} photons cm^{-2} s^{-1}) (all filters, Thorlabs, Ely, UK) (Supplemental material; Figure S1). The light pulses were delivered to the sample via a 10× objective (Olympus, Shinjuku, Japan) mounted to an inverted Zeiss I×71 microscope (Zeiss, Oberkochem, Germany). Stimuli were calibrated using an in-line power meter (Thor Labs, Ely, UK), and values were converted using a validated irradiance conversion toolbox.[87]

Recordings began with 30 s of baseline recording before the onset of a 10 s light pulse. Recording was then continued until 90 s had elapsed. After each recording, an interval of 10 min was allowed for dark adaptation before the next trial.

Statistical analysis

Analysis was carried out using Prism Software version 8.0 (GraphPad, San Diego, CA, USA). Significance was defined as $p < 0.05$. Non-

normality was tested for using the Shapiro-Wilk and Kolmogorov-Smirnov tests. A one-way ANOVA was employed to compare a single variable across groups. With more than one variable, a two-way ANOVA with Tukey's correction for multiple comparisons was used where data was normally distributed and the KW test with Dunn's correction for multiple comparisons when it was not. Values are presented as mean ± standard error of mean.

SUPPLEMENTAL INFORMATION

Supplemental information can be found online at https://doi.org/10.1016/j.omtm.2022.03.003.

ACKNOWLEDGMENTS

The authors tahnk Prof. Hirai, Department of Neurophysiology & Neural Repair, Gunma University Graduate School of Medicine, Japan, for his kind gift of the L7-6 promoter construct and David Smithson, graphic designer, Department of Educational Media, University College London, for assistance with the graphical abstract. This work was supported by The Wellcome Trust (grant number 205151/Z/16/Z to M.J.G.), the Pro Retina Deutschland Foundation (grant number Pro-Re/Projekt/Gilhooley-Whitehead-Lindner.04-2021 to M.J.G. and M.L.), and the University Hospital Gießen and Marburg Research Funds (15/2020MR to M.L.) as well as the Biological Basic Sciences Research Council (grant number BB/M009998/1 to S.H., S.N.P., and M.W.H.). M.W.H. acknowledges the support of the Medical Research Council UK (MR/S026266/1). Preliminary findings from of this research were presented at the 2020 and 2021 annual meetings of the Association for Research in Vision and Ophthalmology (ARVO) and the 2020 and 2021 annual meetings of the North American Neuroophthalmology Society (NANOS).

AUTHOR CONTRIBUTIONS

M.J.G. planned and carried out experiments, analyzed data, and wrote and critically revised the manuscript. M.L. planned and carried out experiments and critically reviewed the manuscript. T.P. carried out experiments and critically reviewed the manuscript. S.H. planned and carried out experiments and critically reviewed the manuscript. S.N.P. planned experiments and critically reviewed the manuscript. M.W.H. planned experiments and critically reviewed the manuscript.

DECLARATION OF INTERESTS

M.L. has received financial support from Heidelberg Engineering, Optos, and Genentech and is an equity owner of Fresenius Medical Care. The other authors declare no competing interests.

REFERENCES

1. Hartong, D.T., Berson, E.L., and Dryja, T.P. (2006). Retinitis pigmentosa. Lancet *368*, 1795–1809.

2. Russell, S., Bennett, J., Wellman, J.A., Chung, D.C., Yu, Z.-F., Tillman, A., Wittes, J., Pappas, J., Elci, O., and McCague, S. (2017). Efficacy and safety of voretigene neparvovec (AAV2-hRPE65v2) in patients with RPE65-mediated inherited retinal dystrophy: a randomised, controlled, open-label, phase 3 trial. Lancet *390*, 849–860.

3. Berry, M.H., Holt, A., Salari, A., Veit, J., Visel, M., Levitz, J., Aghi, K., Gaub, B.M., Sivyer, B., Flannery, J.G., et al. (2019). Restoration of high-sensitivity and adapting vision with a cone opsin. Nat. Commun. *10*, 1221.

4. Bi, A., Cui, J., Ma, Y.-P., Olshevskaya, E., Pu, M., Dizhoor, A.M., and Pan, Z.-H. (2006). Ectopic expression of a microbial-type rhodopsin restores visual responses in mice with photoreceptor degeneration. Neuron *50*, 23–33.

5. Cehajic-Kapetanovic, J., Eleftheriou, C., Allen, A.E., Milosavljevic, N., Pienaar, A., Bedford, R., Davis, K.E., Bishop, P.N., and Lucas, R.J. (2015). Restoration of vision with ectopic expression of human rod opsin. Curr. Biol. *25*, 2111–2122.

6. De Silva, S.R., Barnard, A.R., Hughes, S., Tam, S.K.E., Martin, C., Singh, M.S., Barnea-Cramer, A.O., McClements, M.E., During, M.J., Peirson, S.N., et al. (2017). Long-term restoration of visual function in end-stage retinal degeneration using subretinal human melanopsin gene therapy. Proc. Natl. Acad. Sci. U S A *114*, 11211–11216.

7. Ganjawala, T.H., Lu, Q., Fenner, M.D., Abrams, G.W., and Pan, Z.-H. (2019). Improved channelrhodopsin variants restore visual acuity and contrast sensitivity in A mouse model of blindness under ambient light conditions. Mol. Ther. *27*, 1195–1205.

8. Gaub, B.M., Berry, M.H., Holt, A.E., Isacoff, E.Y., and Flannery, J.G. (2015). Optogenetic vision restoration using rhodopsin for enhanced sensitivity. Mol. Ther. *23*, 1562–1571.

9. Liu, M.M., Dai, J.M., Liu, W.Y., Zhao, C.J., Lin, B., and Yin, Z.Q. (2016). Human melanopsin-AAV2/8 transfection to retina transiently restores visual function in rd1 mice. Int. J. Ophthalmol. *9*, 655–661.

10. Sengupta, A., Chaffiol, A., Macé, E., Caplette, R., Desrosiers, M., Lampič, M., Forster, V., Marre, O., Lin, J.Y., and Sahel, J.A. (2016). Red-shifted channelrhodopsin stimulation restores light responses in blind mice, macaque retina, and human retina. EMBO Mol. Med. *8*, 1248–1264.

11. Thyagarajan, S., van Wyk, M., Lehmann, K., Lowel, S., Feng, G., and Wassle, H. (2010). Visual function in mice with photoreceptor degeneration and transgenic expression of channelrhodopsin 2 in ganglion cells. J. Neurosci. *30*, 8745–8758.

12. van Wyk, M., Pielecka-Fortuna, J., Lowel, S., and Kleinlogel, S. (2015). Restoring the ON switch in blind retinas: opto-mGluR6, a next-generation, cell-tailored optogenetic tool. PLoS Biol. *13*, e1002143.

13. Lindner, M., Gilhooley, M.J., Peirson, S.N., Hughes, S., and Hankins, M.W. (2020). The functional characteristics of optogenetic gene therapy for vision restoration. Cell Mol. Life Sci. *78*, 1597–1613.

14. Lu, Q., and Pan, Z.-H. (2021). Optogenetic strategies for vision restoration. In Optogenetics: Light-Sensing Proteins and Their Applications in Neuroscience and beyond, H. Yawo, H. Kandori, A. Koizumi, and R. Kageyama, eds. (Springer Singapore), pp. 545–555.

15. Simon, C.J., Sahel, J.A., Duebel, J., Herlitze, S., and Dalkara, D. (2020). Opsins for vision restoration. Biochem. Biophys. Res. Commun. *527*, 325–330.

16. Sahel, J.A., Boulanger-Scemama, E., Pagot, C., Arleo, A., Galluppi, F., Martel, J.N., Esposti, S.D., Delaux, A., de Saint Aubert, J.B., de Montleau, C., et al. (2021). Partial recovery of visual function in a blind patient after optogenetic therapy. Nat. Med. *27*, 1223–1229.

17. Macé, E., Caplette, R., Marre, O., Sengupta, A., Chaffiol, A., Barbe, P., Desrosiers, M., Bamberg, E., Sahel, J.-A., and Picaud, S. (2015). Targeting channelrhodopsin-2 to ON-bipolar cells with vitreally administered AAV Restores ON and OFF visual responses in blind mice. Mol. Ther. *23*, 7–16.

18. Lagali, P.S., Balya, D., Awatramani, G.B., Munch, T.A., Kim, D.S., Busskamp, V., Cepko, C.L., and Roska, B. (2008). Light-activated channels targeted to ON bipolar cells restore visual function in retinal degeneration. Nat. Neurosci. *11*, 667–675.

19. Gaub, B.M., Berry, M.H., Holt, A.E., Reiner, A., Kienzler, M.A., Dolgova, N., Nikonov, S., Aguirre, G.D., Beltran, W.A., Flannery, J.G., et al. (2014). Restoration of visual function by expression of a light-gated mammalian ion channel in retinal ganglion cells or ON-bipolar cells. Proc. Natl. Acad. Sci. U S A *111*, E5574–E5583.

20. Lin, B., Koizumi, A., Tanaka, N., Panda, S., and Masland, R.H. (2008). Restoration of visual function in retinal degeneration mice by ectopic expression of melanopsin. Proc. Natl. Acad. Sci. U S A *105*, 16009–16014.

21. Liu, W., Liu, M., Liu, Y., Li, S., Weng, C., Fu, Y., He, J., Gong, Y., Liu, W., Zhao, C., et al. (2018). Validation and Safety of visual restoration by ectopic expression of human melanopsin in retinal ganglion cells. Hum. Gene Ther. *30*, 714–726.

22. Lu, Q., Ganjawala, T.H., Hattar, S., Abrams, G.W., and Pan, Z.H. (2018). A robust optomotor assay for assessing the efficacy of optogenetic tools for vision restoration. Invest. Ophthalmol. Vis. Sci. *59*, 1288–1294.

23. Lu, Q., Ganjawala, T.H., Krstevski, A., Abrams, G.W., and Pan, Z.H. (2020). Comparison of AAV-mediated optogenetic vision restoration between retinal ganglion cell expression and ON bipolar cell targeting. Mol. Ther. Methods Clin. Dev. *18*, 15–23.

24. Hughes, S., Rodgers, J., Hickey, D., Foster, R.G., Peirson, S.N., and Hankins, M.W. (2016). Characterisation of light responses in the retina of mice lacking principle components of rod, cone and melanopsin phototransduction signalling pathways. Sci. Rep. *6*, 28086.

25. Qiu, X., Kumbalasiri, T., Carlson, S.M., Wong, K.Y., Krishna, V., Provencio, I., and Berson, D.M. (2005). Induction of photosensitivity by heterologous expression of melanopsin. Nature *433*, 745–749.

26. Palumaa, T., Gilhooley, M.J., Jagannath, A., Hankins, M.W., Hughes, S., and Peirson, S.N. (2018). Melanopsin: photoreceptors, physiology and potential. Curr. Opin. Physiol. *5*, 68–74.

27. Ivanova, E., Lee, P., and Pan, Z.H. (2013). Characterization of multiple bistratified retinal ganglion cells in a purkinje cell protein 2-Cre transgenic mouse line. J. Comp. Neurol. *521*, 2165–2180.

28. Ivanova, E., Hwang, G.S., and Pan, Z.H. (2010). Characterization of transgenic mouse lines expressing Cre recombinase in the retina. Neuroscience *165*, 233–243.

29. Kleinlogel, S., Vogl, C., Jeschke, M., Neef, J., and Moser, T. (2020). Emerging approaches for restoration of hearing and vision. Physiol. Rev. *100*, 1467–1525.

30. Hulliger, E.C., Hostettler, S.M., and Kleinlogel, S. (2020). Empowering retinal gene therapy with a specific promoter for human rod and cone ON-bipolar cells. Mol. Ther. Methods Clin. Dev. *17*, 505–519.

31. Wu, Z., Yang, H., and Colosi, P. (2010). Effect of genome size on AAV vector packaging. Mol. Ther. *18*, 80–86.

32. Nitta, K., Matsuzaki, Y., Konno, A., and Hirai, H. (2017). Minimal purkinje cell-specific PCP2/L7 promoter virally available for rodents and non-human primates. Mol. Ther. Methods Clin. Dev. *6*, 159–170.

33. Bailes, H.J., and Lucas, R.J. (2013). Human melanopsin forms a pigment maximally sensitive to blue light (lambdamax approximately 479 nm) supporting activation of G(q/11) and G(i/o) signalling cascades. Proc. Biol. Sci. *280*, 20122987.

34. Spoida, K., Eickelbeck, D., Karapinar, R., Eckhardt, T., Mark, M.D., Jancke, D., Ehinger, B.V., Konig, P., Dalkara, D., Herlitze, S., et al. (2016). Melanopsin variants as intrinsic optogenetic on and off switches for transient versus sustained activation of G protein pathways. Curr. Biol. *26*, 1206–1212.

35. Hughes, S., Jagannath, A., Hickey, D., Gatti, S., Wood, M., Peirson, S.N., Foster, R.G., and Hankins, M.W. (2015). Using siRNA to define functional interactions between melanopsin and multiple G Protein partners. Cell Mol. Life Sci. *72*, 165–179.

36. Hughes, S., Hankins, M.W., Foster, R.G., and Peirson, S.N. (2012). Melanopsin phototransduction: slowly emerging from the dark. Prog. Brain Res. *199*, 19–40.

37. Jiang, Z., Yue, W.W.S., Chen, L., Sheng, Y., and Yau, K.-W. (2018). Cyclic-nucleotide- and HCN-Channel-Mediated phototransduction in intrinsically photosensitive retinal ganglion cells. Cell *175*, 652–664.e12.

38. Mure, L.S., Hatori, M., Zhu, Q., Demas, J., Kim, I.M., Nayak, S.K., and Panda, S. (2016). Melanopsin-encoded response properties of intrinsically photosensitive retinal ganglion cells. Neuron *90*, 1016–1027.

39. Van Gelder, R.N., and Buhr, E.D. (2016). Melanopsin: the tale of the tail. Neuron *90*, 909–911.

40. Blasic, J.R., Jr., Matos-Cruz, V., Ujla, D., Cameron, E.G., Hattar, S., Halpern, M.E., and Robinson, P.R. (2014). Identification of critical phosphorylation sites on the carboxy tail of melanopsin. Biochemistry *53*, 2644–2649.

41. Somasundaram, P., Wyrick, G.R., Fernandez, D.C., Ghahari, A., Pinhal, C.M., Simmonds Richardson, M., Rupp, A.C., Cui, L., Wu, Z., Brown, R.L., et al. (2017). C-terminal phosphorylation regulates the kinetics of a subset of melanopsin-mediated behaviors in mice. Proc. Natl. Acad. Sci. U S A *114*, 2741–2746.

42. Sexton, T.J., and Van Gelder, R.N. (2015). G-protein coupled receptor kinase 2 minimally regulates melanopsin activity in intrinsically photosensitive retinal ganglion cells. PLoS One *10*, e0128690.

43. Cameron, E.G., and Robinson, P.R. (2014). β-Arrestin-dependent deactivation of mouse melanopsin. PLoS One *9*, e113138.

44. Mure, L.S., Hatori, M., Ruda, K., Benegiamo, G., Demas, J., and Panda, S. (2018). Sustained melanopsin photoresponse is supported by specific roles of beta-arrestin 1 and 2 in deactivation and regeneration of photopigment. Cell Rep. *25*, 2497–2509.e4.

45. Panda, S., Nayak, S.K., Campo, B., Walker, J.R., Hogenesch, J.B., and Jegla, T. (2005). Illumination of the melanopsin signaling pathway. Science *307*, 600–604.

46. Haverkamp, S., and Wässle, H. (2000). Immunocytochemical analysis of the mouse retina. J. Comp. Neurol. *424*, 1–23.

47. Nawy, S. (2014). Modulation of TRPM1 and the mGluR6 cascade in ON bipolar cells. Spr Ser. Vis. Res. *3*, 99–119.

48. Ruether, K., Feigenspan, A., Pirngruber, J., Leitges, M., Baehr, W., and Strauss, O. (2010). PKC{alpha} is essential for the proper activation and termination of rod bipolar cell response. Invest. Ophthalmol. Vis. Sci. *51*, 6051–6058.

49. Sulaiman, P., Fina, M., Feddersen, R., and Vardi, N. (2010). Ret-PCP2 colocalizes with protein kinase C in a subset of primate ON cone bipolar cells. J. Comp. Neurol. *518*, 1098–1112.

50. Xiong, W.H., Pang, J.J., Pennesi, M.E., Duvoisin, R.M., Wu, S.M., and Morgans, C.W. (2015). The effect of PKCalpha on the light response of rod bipolar cells in the mouse retina. Invest. Ophthalmol. Vis. Sci. *56*, 4961–4974.

51. Martemyanov, K.A., and Sampath, A.P. (2017). The transduction cascade in retinal ON-bipolar cells: signal processing and disease. Annu. Rev. Vis. Sci. *3*, 25–51.

52. Kralik, J., and Kleinlogel, S. (2021). Functional availability of ON-bipolar cells in the degenerated retina: timing and longevity of an optogenetic gene therapy. Int. J. Mol. Sci. *22*, 11515.

53. Kleinlogel, S., Feldbauer, K., Dempski, R.E., Fotis, H., Wood, P.G., Bamann, C., and Bamberg, E. (2011). Ultra light-sensitive and fast neuronal activation with the Ca(2)+-permeable channelrhodopsin CatCh. Nat. Neurosci. *14*, 513–518.

54. Feldbauer, K., Zimmermann, D., Pintschovius, V., Spitz, J., Bamann, C., and Bamberg, E. (2009). Channelrhodopsin-2 is a leaky proton pump. Proc. Natl. Acad. Sci. U S A *106*, 12317–12322.

55. Miyashita, T., Shao, Y.R., Chung, J., Pourzia, O., and Feldman, D.E. (2013). Long-term channelrhodopsin-2 (ChR2) expression can induce abnormal axonal morphology and targeting in cerebral cortex. Front. Neural Circuits *7*, 8.

56. Maimon, B.E., Diaz, M., Revol, E.C.M., Schneider, A.M., Leaker, B., Varela, C.E., Srinivasan, S., Weber, M.B., and Herr, H.M. (2018). Optogenetic peripheral nerve immunogenicity. Sci. Rep. *8*, 14076.

57. Nagel, G., Szellas, T., Huhn, W., Kateriya, S., Adeishvili, N., Berthold, P., Ollig, D., Hegemann, P., and Bamberg, E. (2003). Channelrhodopsin-2, a directly light-gated cation-selective membrane channel. Proc. Natl. Acad. Sci. U S A *100*, 13940–13945.

58. Klapper, S.D., Swiersy, A., Bamberg, E., and Busskamp, V. (2016). Biophysical properties of optogenetic tools and their application for vision restoration approaches. Front. Syst. Neurosci. *10*, 74.

59. Eleftheriou, C.G., Cehajic-Kapetanovic, J., Martial, F.P., Milosavljevic, N., Bedford, R.A., and Lucas, R.J. (2017). Meclofenamic acid improves the signal to noise ratio for visual responses produced by ectopic expression of human rod opsin. Mol. Vis. *23*, 334–345.

60. Juttner, J., Szabo, A., Gross-Scherf, B., Morikawa, R.K., Rompani, S.B., Hantz, P., Szikra, T., Esposti, F., Cowan, C.S., Bharioke, A., et al. (2019). Targeting neuronal and glial cell types with synthetic promoter AAVs in mice, non-human primates and humans. Nat. Neurosci. *22*, 1345–1356.

61. van Wyk, M., Hulliger, E.C., Girod, L., Ebneter, A., and Kleinlogel, S. (2017). Present molecular limitations of ON-bipolar cell targeted gene therapy. Front. Neurosci. *11*, 161.

62. Doroudchi, M.M., Greenberg, K.P., Liu, J., Silka, K.A., Boyden, E.S., Lockridge, J.A., Arman, A.C., Janani, R., Boye, S.E., Boye, S.L., et al. (2011). Virally delivered channelrhodopsin-2 safely and effectively restores visual function in multiple mouse models of blindness. Mol. Ther. *19*, 1220–1229.

63. Cronin, T., Vandenberghe, L.H., Hantz, P., Juttner, J., Reimann, A., Kacso, A.E., Huckfeldt, R.M., Busskamp, V., Kohler, H., Lagali, P.S., et al. (2014). Efficient

transduction and optogenetic stimulation of retinal bipolar cells by a synthetic adeno-associated virus capsid and promoter. EMBO Mol. Med. *6*, 1175–1190.

64. Gilhooley, M.J., Hickey, D.G., Lindner, M., Palumaa, T., Hughes, S., Peirson, S.N., MacLaren, R.E., and Hankins, M.W. (2021). ON-bipolar cell gene expression during retinal degeneration: implications for optogenetic visual restoration. Exp. Eye Res. *207*, 108553.
65. Jones, B.W., and Marc, R.E. (2005). Retinal remodeling during retinal degeneration. Exp. Eye Res. *81*, 123–137.
66. Marc, R.E., Jones, B.W., Watt, C.B., and Strettoi, E. (2003). Neural remodeling in retinal degeneration. Prog. Retin. Eye Res. *22*, 607–655.
67. Kalloniatis, M., Nivison-Smith, L., Chua, J., Acosta, M.L., and Fletcher, E.L. (2016). Using the rd1 mouse to understand functional and anatomical retinal remodelling and treatment implications in retinitis pigmentosa: a review. Exp. Eye Res. *150*, 106–121.
68. Marc, R.E., and Jones, B.W. (2003). Retinal remodeling in inherited photoreceptor degenerations. Mol. Neurobiol. *28*, 139–147.
69. Marc, R.E., Jones, B.W., Anderson, J.R., Kinard, K., Marshak, D.W., Wilson, J.H., Wensel, T., and Lucas, R.J. (2007). Neural reprogramming in retinal degeneration. Invest. Ophthalmol. Vis. Sci. *48*, 3364–3371.
70. Varela, C., Igartua, I., De la Rosa, E.J., and De la Villa, P. (2003). Functional modifications in rod bipolar cells in a mouse model of retinitis pigmentosa. Vis. Res. *43*, 879–885.
71. Dunn, F.A. (2015). Photoreceptor ablation initiates the immediate loss of glutamate receptors in postsynaptic bipolar cells in retina. J. Neurosci. *35*, 2423–2431.
72. Gayet-Primo, J., and Puthussery, T. (2015). Alterations in Kainate receptor and TRPM1 localization in bipolar cells after retinal photoreceptor degeneration. Front. Cell Neurosci. *9*, 486.
73. Korecki, A.J., Cueva-Vargas, J.L., Fornes, O., Agostinone, J., Farkas, R.A., Hickmott, J.W., Lam, S.L., Mathelier, A., Zhou, M., Wasserman, W.W., et al. (2021). Human MiniPromoters for ocular-rAAV expression in ON bipolar, cone, corneal, endothelial, Muller glial, and PAX6 cells. Gene Ther. *28*, 351–372.
74. de Leeuw, C.N., Korecki, A.J., Berry, G.E., Hickmott, J.W., Lam, S.L., Lengyell, T.C., Bonaguro, R.J., Borretta, L.J., Chopra, V., Chou, A.Y., et al. (2016). rAAV-compatible MiniPromoters for restricted expression in the brain and eye. Mol. Brain *9*, 52.
75. de Leeuw, C.N., Dyka, F.M., Boye, S.L., Laprise, S., Zhou, M., Chou, A.Y., Borretta, L., McInerny, S.C., Banks, K.G., Portales-Casamar, E., et al. (2014). Targeted CNS delivery using human MiniPromoters and demonstrated compatibility with adeno-associated viral vectors. Mol. Ther. Methods Clin. Dev. *1*, 5.
76. Portales-Casamar, E., Swanson, D.J., Liu, L., de Leeuw, C.N., Banks, K.G., Ho Sui, S.J., Fulton, D.L., Ali, J., Amirabbasi, M., Arenillas, D.J., et al. (2010). A regulatory toolbox of MiniPromoters to drive selective expression in the brain. Proc. Natl. Acad. Sci. U S A *107*, 16589–16594.
77. Lu, Q., Ganjawala, T.H., Ivanova, E., Cheng, J.G., Troilo, D., and Pan, Z.H. (2016). AAV-mediated transduction and targeting of retinal bipolar cells with improved mGluR6 promoters in rodents and primates. Gene Ther. *23*, 680–689.
78. Baden, T., Berens, P., Franke, K., Roman Roson, M., Bethge, M., and Euler, T. (2016). The functional diversity of retinal ganglion cells in the mouse. Nature *529*, 345–350.
79. Bowes, C., Li, T., Danciger, M., Baxter, L.C., Applebury, M.L., and Farber, D.B. (1990). Retinal degeneration in the rd mouse is caused by a defect in the β subunit of rod cGMP-phosphodiesterase. Nature *347*, 677.
80. Hattar, S., Liao, H.W., Takao, M., Berson, D.M., and Yau, K.W. (2002). Melanopsin-containing retinal ganglion cells: architecture, projections, and intrinsic photosensitivity. Science *295*, 1065–1070.
81. Silvia Marino, P.K., Leung, C., Hetty, A., van der Korput, G.M., Jan, T., Camenisch, I., Berns, A., and Brandner, S. (2002). PTEN is essential for cell migration but not for fate determination and tumourigenesis in the cerebellum. Development *129*, 3513–3522.
82. Lu, Q., Ivanova, E., Ganjawala, T.H., and Pan, Z.H. (2013). Cre-mediated recombination efficiency and transgene expression patterns of three retinal bipolar cell-expressing Cre transgenic mouse lines. Mol. Vis. *19*, 1310–1320.
83. Nakazawa, K., Quirk, M.C., Chitwood, R.A., Watanabe, M., Yeckel, M.F., Sun, L.D., Kato, A., Carr, C.A., Johnston, D., and Wilson, M.A. (2002). Requirement for hippocampal CA3 NMDA receptors in associative memory recall. Science *297*, 211–218.
84. de Silva, S.R., McClements, M.E., Hankins, M.W., and MacLaren, R.E. (2015). Adeno-associated viral gene therapy for retinal disorders. Gene Deliv. Ther. Neurol. Disord. 203–228.
85. Lin, J.Y., Knutsen, P.M., Muller, A., Kleinfeld, D., and Tsien, R.Y. (2013). ReaChR: a red-shifted variant of channelrhodopsin enables deep transcranial optogenetic excitation. Nat. Neurosci. *16*, 1499–1508.
86. Schindelin, J., Arganda-Carreras, I., Frise, E., Kaynig, V., Longair, M., Pietzsch, T., Preibisch, S., Rueden, C., Saalfeld, S., and Schmid, B. (2012). Fiji: an open-source platform for biological-image analysis. Nat. Methods *9*, 676.
87. Lucas, R.J., Peirson, S.N., Berson, D.M., Brown, T.M., Cooper, H.M., Czeisler, C.A., et al. (2014). Measuring and using light in the melanopsin age. Trends Neurosci. *37*, 1–9.

OMTM, Volume *25*

Supplemental information

A systematic comparison of optogenetic approaches to visual restoration

Michael J. Gilhooley, Moritz Lindner, Teele Palumaa, Steven Hughes, Stuart N. Peirson, and Mark W. Hankins

Supplemental Materials

Methods

Mice

The following origin lines were bred together to reach the desired genotypes.

Table S1 - Genetic background of the various origin lines contributing to the two multi-transgenic lines described. The righthand column refers to genes in which mutations are commonly found in the background strain – not necessarily the particular line used

Origin Line	Genetic Background Strain	Common visually significant mutations[1]
$Pde6b^{rd1/rd12}$[4]	C3H/HeN	$Gpr179^{nob/nob}$, $Pde6b^{rd1/rd1}$
$Opn4^{tm1Yau}$[5]	C57BL/6J	Normal vision
Tg(L7-Cre)[6]	FVB	$Pde6b^{rd1/rd1}$
Tg(grk4-Cre)[7]	C57BL/6J	Normal vision

Experimental mice were of the genotype:

1. Tg(*L7.Cre*)$^{+/-}$; $Pde6b^{rd1/rd1}$; $Opn4^{tm1yau/tm1yau}$
2. Tg(*Grk4.Cre*)$^{+/-}$; $Pde6b^{rd1/rd1}$; $Opn4^{tm1yau/tm1yau}$

As the insertion locus of the Cre transgene in both lines is not known, breeding pairs were maintained as hemizygous x wild type pairs to ensure hemizygous offspring. All mice were genotyped to ensure they were wild type for the endemic $Gpr179^{nob/nob}$ mutation.

Genotyping

Genotypes of individual mice were determined using polymerase chain reaction (PCR) protocols. DNA was extracted from mouse ear clips using sodium hydroxide solution, neutralised with Trizma - HCl. Samples were then incubated with a PCR master mix and primers as described in Table S2 according to manufacturer's instructions. Protocols using Immored® (Bioline, London, UK) ($Pde6b^{rd1}$, *Tg(Cre)*, $Gpr179^{nob1}$) were then run on a 2% agarose gel incorporating 4 μmol/l ethidium bromide (Sigma Aldrich, Gillingham, UK) in Tris-acetate-EDTA (TEA) buffer (Severn Biotech, Kidderminster, UK) at 125volts for 25 minutes. SYBR Green® protocols ($Opn4^{+}$ & $Opn4^{tm1Yau}$ (LacZ)) underwent a melt curve protocol with the characteristic product melt temperature used to confirm the amplification of the specific product.

Confirmation of the retinal phenotype (IHC)

The expected retinal degenerate phenotype was confirmed on microscopy of retinal sections, demonstrating an absence of photoreceptor layer in both lines. The expected $Opn4^{-/-}$ phenotype was confirmed by replacement of mouse OPN4 immunoreactivity in the ganglion cell layer with immunoreactivity for β-galactosidase (encoded by *LacZ*) in the same distribution. Cre recombinase

activity allowed appropriate expression of floxed viral protein in both the L7.Cre and Grk4.Cre model (see Figure 1); confirming the expected Cre phenotype.

Table S2 Primers and reaction parameters for polymerase chain reaction based genotyping of mice.

Allele	Primer	Sequence	Product size or product melt tempterature Tm (bp or °C)	
Pde6b rd1	1	TGACAATTACTCCTTTTCCCTCAGTCT		
	2	GTAAACAGCAAGAGGCTTTATTGGGAA	*Pde6b* rd1	550
	3	GCATTAATTCTGGGGCGCATG	*Pde6b* + (wildtype)	400
Tg(Cre)	F	GCGGTCTGGCAGTAAAAACTATC		
	R	GTGAAACAGCATTGCTGTCACTT		
	F (control)	CGTAGGCCACAGAATTGAAAGATCT	Tg(Cre)	101
	R (control)	GTAGGTGGAAATTCTAGCATCATCC	Control	324
Gpr179 nob	1	TGTGCCTGGGTATCTGTTGA		
	2	GCATGTGCCAAGGGTATCTT	*Gpr179* nob	400
	3	GCTTACACACTTACACACAGATAGATG	*Gpr179* + (wildtype)	113
Opn4 tm1Yau (LacZ)	F	AAGCAGTCAGCAGCCCAAAG		
	R	TGTCACCCTCCTGGTCTTGC	*Opn4* tm1Yau (LacZ)	86°C
Opn4 +	F	AGAAGTGGCTTTGGGGA		
	R	GCAGAAGGCATAGAACTCG	*Opn4* +	80.2°C

AAV production

The CBA.hOPN4 and (floxed) EF1α-hOPN4 construct plasmids were produced in our laboratory as has been described previously[8, 9]. The (floxed) hSyn.DIO.ReaChR construct was a kind gift from Roger Tsein (R.I.P.) (Addgene plasmid # 50954; http://n2t.net/addgene:50954 ; RRID:Addgene_50954) [10]. These plasmids were used, along with a quad mutant capsid (Y-F)[11] and helper (pAdDF6) plasmids to produce adenoassociated virus according to previously published methods[12].

The titre of DNase1-treated virus was determined by real-time quantitative PCR using the standard curve method designed to amplify a portion of the inverted terminal repeat sequence (ITR) as previously described (Primers: forward: 5'-GGA ACC CCT AGT GAT GGA GTT; reverse: 5'-CGG CCT CAG TGA GCG A)[13].

Intravitreal injections

Anaesthesia was induced and maintained in mice via inhalation of isoflurane (Zoetis, Walton Oaks, UK), Guttatae (gt.) 0.5% proxymethocaine & gt. 1% tropicamide (Minnims brand, Bausch & Lomb U.K Limited, Kingston-Upon-Thames) were applied for topical anaesthesia and to dilate the pupil. Gt. Povidone Iodine 5% (Minims brand, Bausch & Lomb U.K Limited, Kingston-Upon-Thames) was added, before Viscotears (Bausch & Lomb U.K Limited, Kingston-Upon-Thames) and a 2mm round cover slip were applied to the cornea view the posterior segment. A 35-gauge, bevelled needle (NF35BV), on a 10μl NanoFil syringe (both World Precision Instruments, Hitchin, UK) was introduced anterior to the gripping forceps. The needle was advanced into the vitreous, under direct visualisation and 1.5μl of undiluted virus (CBA.hOPN4 $4.43x10^{12}$ viral genomes ml^{-1}, floxed.hOPN4 $1.72x10^{12}$ $vgml^{-1}$, floxed.ReaChR $1.5x10^{13}$ $vgml^{-1}$, L7-6.hOPN4 $7.33x10^{12} vgml^{-1}$) or 0.9% saline was then injected. The needle was held in place for 5 to 10 seconds before a slow withdrawal in order to minimise reflux.

Tissue collection & Immunohistochemistry of retinal flat mounts

Animals were culled by cervical dislocation, eyes immediately enucleated and transferred to 4% PFA for twenty-four hours before dissection under a microscope (EZ4D, Leica, Wetzlar, Germany. Permeabilisation was with Triton X-100 1% in PBS, followed by three ten-minute washes in PBS. A blocking step followed with 10% serum from the animal in which the secondary antibody was raised (Table S4) diluted in 1% Triton X-100 for sixty minutes. Slides were then incubated for three days with a primary antibody solution at the stated concentration (Table S3) diluted in 1% Triton X-100 with

2.5% serum from the animal in which the secondary antibody was raised (Table S4). Three washes of thirty minutes in 0.1% Tween-20 were undertaken before incubation overnight with a secondary antibody solution at a concentration of 1:250 in 1% Triton X-100 - PBS (Table S4). Three washes of thirty minutes in 0.1% Tween-20 were undertaken, followed by a 30-minute incubation with 1:30,000 (circa. 300nmol/l) 4',6-Diamidino-2-Phenylindole, Dihydrochloride (DAPI) (ThermoFisher, Loughborough,UK) and a final wash for 30 minutes in ddH_2O. Slides were then dried and mounted with ProLong® Diamond mounting media (ThermoFisher, Loughborough, UK).
Flat mounts were visualised using a confocal microscope (LSM710; Zeiss, Oberkochen, Germany). Whole retina views of flat mounts were achieved by stitching smaller, overlapping images. Images were then processed to using Fiji-ImageJ®[14].

Table S3 - Primary antibodies used

Target	Target species	Manufacturer	Product Code	Host Animal	Dilution used
Melanopsin	Mouse	Advanced Targeting Systems	uf006 - UF006	Rabbit	1:1,000
Melanopsin	Human	Santa Cruz	sc32870	Rabbit	1:1,000
Green Fluorescent Protein	n/a	Aves	gfp1020	Chicken	1:1000
β-galactasdase	n/a	Abcam	ab9361	Chicken	1:500
L7	Mouse	Santa Cruz	sc137064	Mouse	1:1,000

Table S4 - Secondary antibodies used

Name	Target species	Host Animal	Excitation λ (nm)	Emission λ (nm)	Manufacturer	Product Code	Dilution used
Alexa®-568	Rabbit	Donkey	578	603	invitrogen	a10042	1:250
Alexa®-568	Chicken	Goat	578	603	invitrogen	ag175472	1:250
Alexa®-568	Mouse	Donkey	578	603	invitrogen	a10037	1:250
Alexa®-488	Rabbit	Donkey	495	519	invitrogen	a21206	1:250
Alexa®-488	Mouse	Donkey	495	519	invitrogen	a2102	1:250
Alexa®-488	Chicken	Donkey	495	519	Jackson Immunoresearch	T03-545-155	1:250
DAPI	n/a	n/a	358	461	Thermofisher Scientific	D1306	$300nmoll^{-1}$

Multiple Electrode Array Analysis

Retinal ganglion cell action potential firing rate was extracted in 1 second bins using the MC Rack software from filtered data, with an action potential spike defined as a deviation greater than three standard deviations from the mean baseline noise. This data was then transferred to an Excel spreadsheet template (Modified from the work of S Hughes[15]) for further processing.

Proportion of electrodes showing light responses

Table S5 - Proportion of responsive electrodes; Supplemental to figure 2c

Group	CBA.hOPN4	L7.hOPN4	Grik4.hOPN4	L7.ReaChR	Grik4.hOPN4	L7 Saline	Grik4 Saline
Retinae (N=)	5	6	6	11	5	8	8
Responsive (n=)	55	84	87	92	75	7*	6*
Total	300	360	360	660	300	480	480
Proportion	18%	23%	24%	14%	25%	1%	1%

A light responsive electrode was defined as one with a mean rise of 10Hz in spike firing rate, maintained for 10 seconds (rolling 10 second bins) when compared to mean baseline firing rate in the 10 seconds preceding the stimulus (Table S5). Unresponsive electrodes were excluded from further analysis. In the saline sham injection control group, 1% of electrodes were scored as responsive by this metric: all seven of these individual records were manually reviewed (5 of them being adjacent electrodes on the same retina) and did not represent light responses, but spurious readings (noise), that were not present of repeat trials and were so excluded from further analysis. Similarly, six electrodes (five adjacent on the same retina) were scored responsive in the saline treated Grk4.Cre group but seen on manual review to similarly represent noise and were excluded from analysis.

Irradiance Response Curve Fitting

Responses (maximum firing rate post stimulus) at each of 7 intensities (circa 10^{10} to 10^{16} photons cm^{-2} s^{-1}) were normalised to the response at the intensity with the largest response. A macro, written in visual basic and using MS® Excel's solver module (modified from the work of S Hughes & J Rodgers[9, 15, 16]) using an iterative non-linear regression process, was used to fit a sigmoidal curve to this normalised data by minimising the sum of squares of the regression[17]. The Hill Slope & EC_{50} value was allowed to vary in order to achieve the best fit [18]. Starting values in both cases were defined as an EC_{50} = 13 and Hill slope = 1, as plausible values for photopigments[18]. In both cases, only electrodes where a fit of r^2>0.8 were included in reported mean EC_{50} or used in comparison of curves between groups in the main manuscript. IRC plots of Individual EC50 and Hill slope values for each electrode are shown in figure S1.

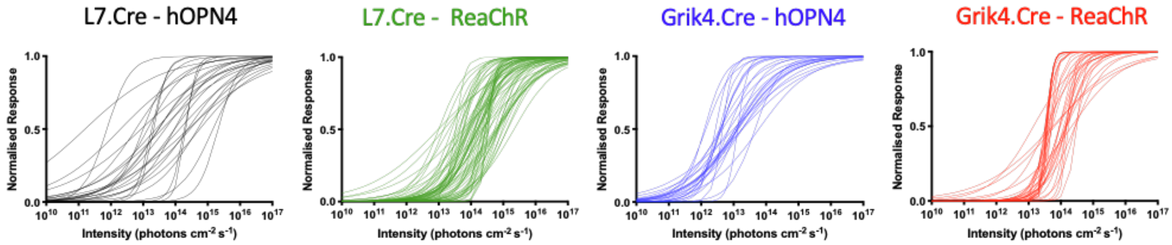

Figure 1 (supplementary) - Plots of IRCs fitted to individual electrodes plotted per treatment group.

Response Decay kinetics

Response decay kinetics, recorded as the total response half life, $t_{1/2}$, was taken as the time interval between the maximum spike firing rate above baseline recorded within 10 seconds of stimulus onset and the point at which the spike firing rate was half this value. Sub-indices were also analysed: spike adaptation half-life was taken as the time interval between the maximum spike firing rate recorded while the stimulus was present and the point at which the firing rate was halfway between this value and that at stimulus offset. True offset half-life was calculated as the time interval between the firing rate at stimulus offset and the time at which the firing rate decayed to half this level. Only values from responsive electrodes with IRC fits r^2>0.8 were included in the mean values that were compared between groups.

Supplemental References

1. Peirson, SN, Brown, LA, Pothecary, CA, Benson, LA, and Fisk, AS (2017). Light and the laboratory mouse. *J Neurosci Methods*.
2. KEELER, C (1966). Retinal degeneration in the mouse is rodless retina. *Journal of Heredity* **57**: 47-50.
3. Keeler, CE (1924). The inheritance of a retinal abnormality in white mice. *Proceedings of the National Academy of Sciences of the United States of America* **10**: 329.
4. Pittler, SJ, and Baehr, W (1991). Identification of a nonsense mutation in the rod photoreceptor cGMP phosphodiesterase beta-subunit gene of the rd mouse. *Proc Natl Acad Sci U S A* **88**: 8322-8326.
5. Hattar, S, Liao, HW, Takao, M, Berson, DM, and Yau, KW (2002). Melanopsin-containing retinal ganglion cells: architecture, projections, and intrinsic photosensitivity. *Science* **295**: 1065-1070.
6. Silvia Marino, PK, Carly Leung, Hetty A. G. M. van der Korput, Jan Trapman, Isabelle Camenisch, Anton Berns and Sebastian Brandner (2002). PTEN is essential for cell migration but not for fate determination and tumourigenesis in the cerebellum. *Development* **129**: 3513-3522.
7. Nakazawa, K, Quirk, MC, Chitwood, RA, Watanabe, M, Yeckel, MF, Sun, LD*, et al.* (2002). Requirement for hippocampal CA3 NMDA receptors in associative memory recall. *Science* **297**: 211-218.
8. De Silva, SR, Barnard, AR, Hughes, S, Tam, SKE, Martin, C, Singh, MS*, et al.* (2017). Long-term restoration of visual function in end-stage retinal degeneration using subretinal human melanopsin gene therapy. *Proc Natl Acad Sci U S A* **114**: 11211-11216.
9. Rodgers, J, Hughes, S, Pothecary, CA, Brown, LA, Hickey, DG, Peirson, SN*, et al.* (2018). Defining the impact of melanopsin missense polymorphisms using in vivo functional rescue. *Hum Mol Genet*.
10. Lin, JY, Knutsen, PM, Muller, A, Kleinfeld, D, and Tsien, RY (2013). ReaChR: a red-shifted variant of channelrhodopsin enables deep transcranial optogenetic excitation. *Nature neuroscience* **16**: 1499-1508.
11. Petrs-Silva, H, Dinculescu, A, Li, Q, Min, SH, Chiodo, V, Pang, JJ*, et al.* (2009). High-efficiency transduction of the mouse retina by tyrosine-mutant AAV serotype vectors. *Mol Ther* **17**: 463-471.
12. de Silva, SR, McClements, ME, Hankins, MW, and MacLaren, RE (2015). Adeno-Associated Viral Gene Therapy for Retinal Disorders. *Gene Delivery and Therapy for Neurological Disorders*: 203-228.
13. Aurnhammer, C, Haase, M, Muether, N, Hausl, M, Rauschhuber, C, Huber, I*, et al.* (2012). Universal real-time PCR for the detection and quantification of adeno-associated virus serotype 2-derived inverted terminal repeat sequences. *Hum Gene Ther Methods* **23**: 18-28.
14. Schindelin, J, Arganda-Carreras, I, Frise, E, Kaynig, V, Longair, M, Pietzsch, T*, et al.* (2012). Fiji: an open-source platform for biological-image analysis. *Nature methods* **9**: 676.

15. Hughes, S (2017). An automaded program for MEA analysis written in MS Visual Basic & MS Excel.
16. Kemmer, G, and Keller, S (2010). Nonlinear least-squares data fitting in Excel spreadsheets. *Nat Protoc* **5**: 267-281.
17. Peirson, SN, Thompson, S, Hankins, MW, and Foster, RG (2005). Mammalian photoentrainment: results, methods, and approaches. *Methods in enzymology*, vol. 393. Elsevier. pp 697-726.
18. Govardovskii, VI, Fyhrquist, N, Reuter, TOM, Kuzmin, DG, and Donner, K (2000). In search of the visual pigment template. *Visual Neuroscience* **17**: 509-528.
